# Dynamic Behavior and Substrate Interactions of the Polymyxin Resistance Determinant MCR‑1 Investigated by Molecular Dynamics Simulations in the Membrane Environment

**DOI:** 10.1021/acs.jcim.5c01338

**Published:** 2025-07-22

**Authors:** Emily Lythell, Jack Badley, Reynier Suardíaz, Catherine R. Gurr, Catherine L. Tooke, Philip Hinchliffe, A. Sofia F. Oliveira, Marc W. Van der Kamp, James Spencer, Adrian J. Mulholland

**Affiliations:** † Centre for Computational Chemistry, School of Chemistry, 1980 University of Bristol, Cantock’s Close, Bristol BS8 1TS, U.K.; ‡ School of Cellular and Molecular Medicine, University of Bristol, University Walk, Bristol BS8 1TD, U.K.; § Departamento de Química Física, Facultad de Química, Universidad Complutense, Madrid 28040, Spain; ∥ School of Biochemistry, University of Bristol, University Walk, Bristol BS8 1TD, U.K.; ⊥ Department of Life Sciences, University of Bath, Claverton Down, Bath BA2 7AY, U.K.

## Abstract

The Mobile Colistin Resistance (MCR) phosphoethanolamine (PEtN) transferase is a plasmid-borne enzyme responsible for colistin antibiotic resistance in , the most important antimicrobial-resistant bacterial pathogen worldwide. Bacterial PEtN transferases like MCR comprise periplasmic catalytic and integral membrane domains, with mechanistic understanding largely based on studies of the former and limited information on the full-length enzyme. Previous investigations of a PEtN transferase identified that the catalytic domain can effectively dissociate from the transmembrane component and instead make extensive contacts with the membrane surface. Here, we report molecular dynamics simulations of a model of full-length MCR-1 in a representative membrane comprising 80% of a PEtN donor substrate, palmitoyloleoyl phosphoethanolamine (POPE), that explore the dynamic behavior of the enzyme and the impact upon it of zinc stoichiometry and PEtN addition to the Thr285 acceptor residue. The results identify only limited movement of the two domains relative to one another, and that POPE can bind the likely “resting” state of the enzyme (monozinc with unmodified Thr285) in an orientation compatible with PEtN transfer to Thr285. Stable binding of a second zinc equivalent occurred only with application of restraints and involved Glu116 from the transmembrane domain. Mutation of this residue abolished MCR-1-mediated protection of recombinant from colistin. Our data suggest domain motions in bacterial PEtN transferases to be condition-dependent and support a proposed “ping-pong” reaction mechanism, with the monozinc enzyme competent to undertake the first stage.

## Graphical abstract

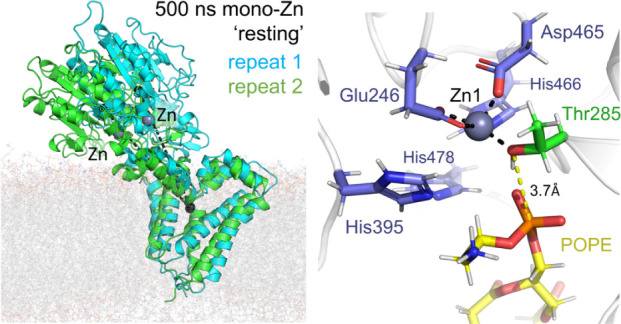

## Introduction

Bacterial antimicrobial resistance (AMR) is a key global public health challenge. Estimates based on 2019 data^1^ identify 1.3 million deaths worldwide as directly attributable to AMR, with AMR being a contributing factor to a further 3.7 million. A previous study^2^ projected an annual total of 10 million deaths by 2050 in the absence of effective interventions. Two species of Enterobacterales, and , are respectively the first and third most common pathogens responsible for AMR-associated deaths. Strains of these organisms resistant to carbapenems or extended-spectrum oxyimino-cephalosporins are considered by the World Health Organization to be critical priority pathogens for antimicrobial research and development^3^ due to the weakness of the antibacterial discovery pipeline for these and other multiresistant Gram-negative bacteria.

Polymyxin antibiotics, specifically colistin, are agents of last resort for treating infections caused by these species^4^ and, despite toxicity concerns,^5^ their use has increased in recent years.^6^ Polymyxins are lipopeptides that disrupt the Gram-negative envelope by a sequential process that remains incompletely understood:^7^ initial interaction with outer membrane lipopolysaccharide (LPS) is followed by uptake into the bacterial periplasm and effects upon the inner membrane that may include interference with LPS before or during trafficking to the outer membrane, and/or direct permeabilization. Polymyxin binding to LPS initially involves electrostatic interactions of positively charged l-α-γ-diaminobutyric acid (Dab) side chains of the polymyxin cyclic heptapeptide headgroup with the negatively charged phosphate groups at the 1’ or 4’ positions on the LPS lipid A moiety, so displacing divalent cations (Ca^2+^, Mg^2+^) that stabilize LPS-containing membranes by facilitating electrostatic interactions between adjacent LPS molecules (for review, see ref 8. Consequently, modifications to LPS that disrupt polymyxin interactions are determinants of polymyxin resistance; specifically, these reduce accessible negative charge on the lipid A disaccharide by covalent modification of phosphate groups, thereby preventing electrostatic interactions with cationic components (Dab) of the polymyxin peptide. Such modifications most commonly involve addition of phosphoethanolamine (PEtN) (Scheme 1A) or 4-amino-4-deoxy-l-arabinose (L-Ara-4N), which present positively charged terminal amine groups to the 1’ or 4’ phosphates.^9^

**Scheme 1:**
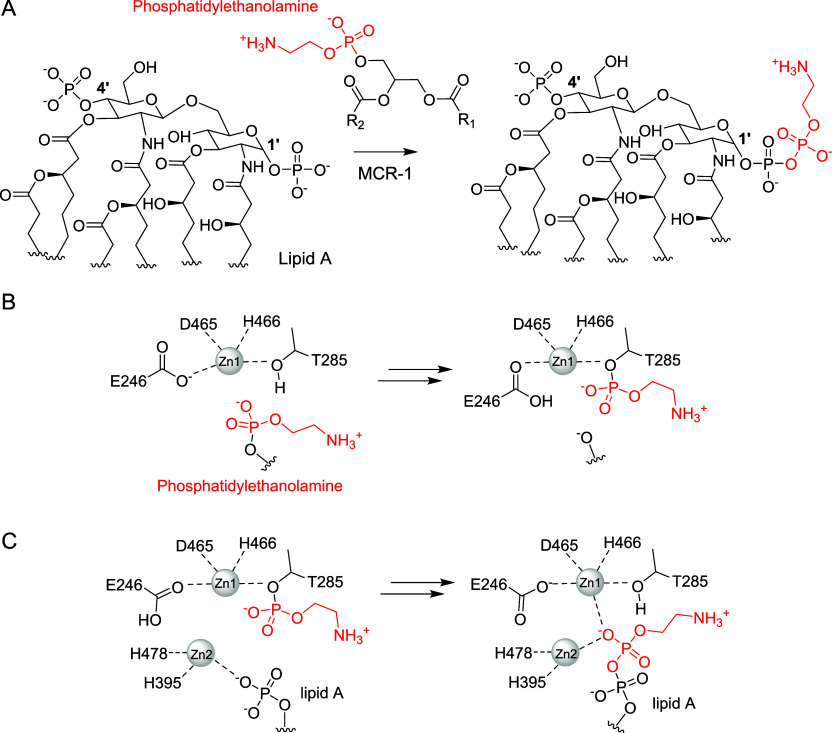
Phosphoethanolamine Transfer to Lipid A Catalyzed by MCR-1.^a^

a (A) Overall reaction involves transfer of phosphoethanolamine to the 1’ (shown) or 4’ phosphate groups of lipid A. This likely occurs via a ping-pong mechanism, involving two distinct steps: (B) First step of the proposed ping-pong reaction in a monozinc active site involves transfer of phosphoethanolamine (red, from a phosphatidyl ethanolamine donor) to Thr285. (C) Second step (proposed to require a dizinc active site) involves transfer of phosphoethanolamine to lipid A, and reprotonation of Thr285. Adapted from Suardíaz et al.^22^

Lipid A phosphoethanolamine transferases are widely distributed in Gram-negative bacteria and are constitutively expressed in some species, including^10^ and ,^11^ that, in consequence, are intrinsically polymyxin-resistant. In other species, including ,^12^ acquired polymyxin resistance can arise from upregulation of chromosomal PEtN transferases, but in this has remained rare. In 2015, however, a plasmid-borne polymyxin resistance determinant, the Mobile Colistin Resistance (MCR)-1 PEtN transferase, was identified in China in an isolate of porcine origin;^13^ since then multiple MCR isoforms have been discovered in multiple species worldwide.^14^ In , MCR production remains the dominant polymyxin resistance mechanism.

Phosphoethanolamine transferases, including MCR, are enzymes of the Gram-negative inner membrane that consist of a soluble periplasmic catalytic domain and a transmembrane domain^15^ (Figure 1). The catalytic domain has been the subject of relatively extensive biochemical study^16–18^ and contains a zinc site (Zn1, comprised of three conserved amino acids: Glu246, Asp465, and His466) adjacent to the threonine residue (Thr285) that is the likely site of formation of the covalent phosphointermediate generated when PEtN is transferred from a phosphatidylethanolamine donor, prior to its addition to the lipid A acceptor substrate (Scheme 1). Two additional conserved “gating” histidine residues (His395 and His478) are observed in some structures of the catalytic domains of MCR-1 (pdbs 5LRM,^16^ 5GOV,^19^ and 5GRR^20^) and of the PEtN transferases from (pdb 4KAY^18^) and (pdb 6BNC^21^) to bind a second zinc equivalent (Zn2). However, co-ordination of this second zinc ion involves either intermolecular interactions with crystallographic dimers^21^ or symmetry-related molecules,^16,19,20^ or (in the enzyme)^18^ an additional undefined ligand. Hence, in the absence of evidence for contributions to zinc coordination from either the bound substrate or the transmembrane domain, the physiological and mechanistic significance of this site remains unclear. The contribution of the transmembrane domain to MCR activity is also incompletely understood, although it is reasonable to assume its involvement in binding both (lipidated) donor and acceptor substrates and potentially in orienting these for PEtN transfer. Given that the majority of studies have investigated the isolated MCR catalytic domain, it is also an open question as to whether residues from the transmembrane domain, in particular Glu116, positioned on a short α-helix that sits on the inner membrane surface, form part of the active site in the intact protein and hence are important in catalyzing the PEtN transfer reaction.

**Figure 1:**
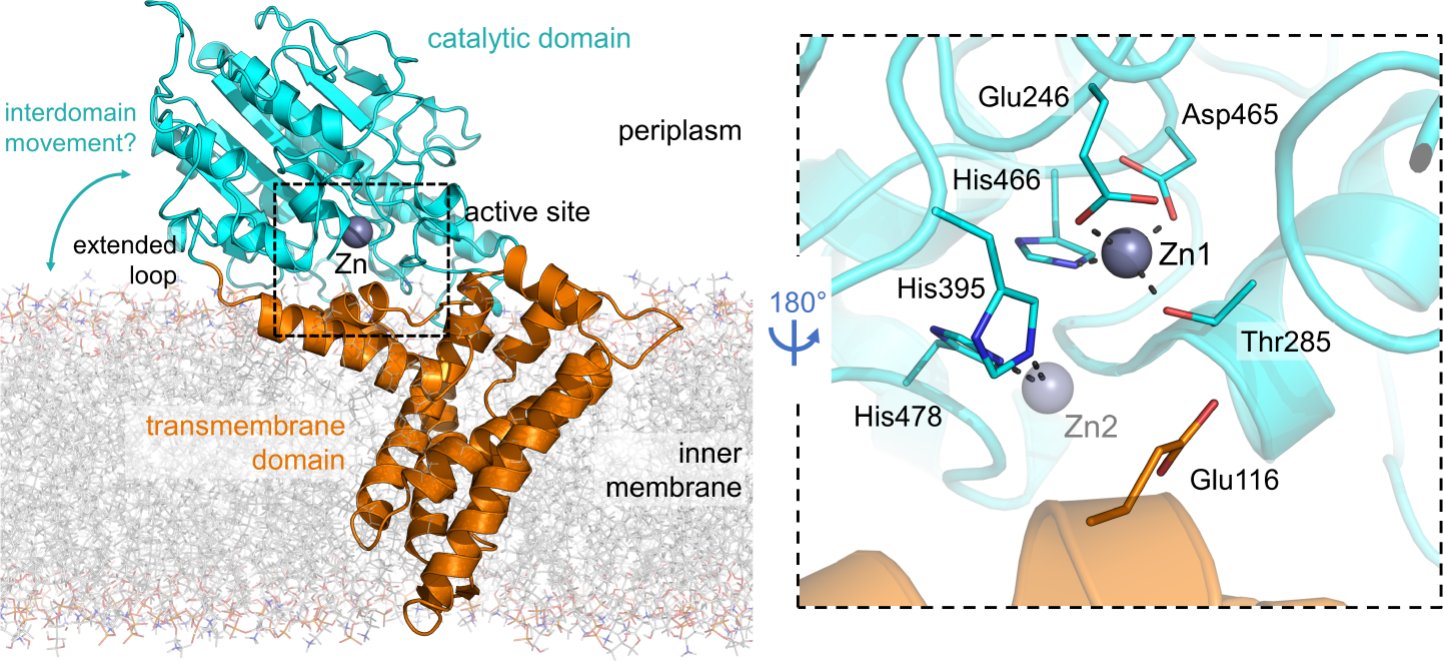
Architecture of MCR-1. Left, overall architecture of MCR-1 (homology model derived from the full-length structure of EptA, PDB 5FGN.^15^ Boxed, active site of MCR-1 based on the crystal structure of the dizinc form of the catalytic domain (PDB 5LRM.^16^ Hydrogen and co-ordination bonds are shown as dashed lines.

To date, an experimentally determined structure of a full-length enzyme, including the transmembrane domain, is only available for one PEtN transferase, the chromosomally encoded EptA enzyme from .^15^ On the basis of this structure, these authors undertook a series of molecular dynamics (MD) simulations of EptA within the membrane environment and in two of six 100 ns trajectories observed the enzyme to undergo a significant conformational change in which the catalytic domain underwent a rotation about the loop connecting the two domains, relative to the transmembrane domain and the membrane, allowing for new membrane contacts and even deformation of the membrane surface. It was hypothesized that this flexibility could allow for entry to the active site of two substrates of differing sizes and so may be important to reactivity of the enzyme. Biophysical studies,^23^ primarily using small-angle X-ray scattering (SAXS), were considered to support this contention by identifying differences in the conformation of EptA in micelles of two different detergents, *n*-dodecyl-β-d-maltoside (DDM) and *n*-dodecyl-phosphocholine (DPC), with the latter promoting a more open, extended organization. To date, however, no such investigations have been reported for any MCR enzyme.

Accordingly, and building on our previous computational investigations of the isolated MCR catalytic domain, we here sought to explore the conformational dynamics of the complete MCR protein using extended (500 ns) molecular dynamics simulations of a homology model in a model membrane environment, in order to investigate interactions with the phosphatidylethanolamine donor substrate and zinc cofactor in the ground (reactive) state, and the effect upon these of PEtN addition to Thr285 to form the covalent phosphointermediate. The results identify only limited motions of the two domains relative to one another and do not support stable binding of a second zinc equivalent under the conditions tested, while establishing that the complex of a donor substrate with the ground state enzyme is capable of sampling a stable and potentially reactive conformation.

## Materials and Methods

### Model Construction

A homology model of full-length MCR-1 (Figure S1) was created using Modeler v. 9.17,^24^ using the EptA^15^ structure (PDB 5FGN, 35.6% sequence identity) as a template. (This model aligns closely with the equivalent model generated using AlphaFold 3 ^25^ (Figure S1)). To create a dizinc starting structure, the second zinc equivalent was positioned by aligning this model with the structure of the MCR-1 catalytic domain in the dizinc form (PDB 5LRM^16^). The final model comprises MCR-1 residues 11–541; the first ten residues were excluded as these form an unstructured N-terminal tail far from the active site and have no predicted structural or catalytic function. Phosphorylated Thr285, as found in the structure of MCR-1 in the monozinc form (PDB 5LRN^16^), was modified to threonine and to phosphoethanolaminated threonine (PET) using the builder tool in PyMOL^26^ to replicate the resting and phosphointermediate states, respectively. The side chain of the phosphoethanolaminated threonine (PET) residue was positioned to avoid side chain clashes with residues from either domain. The engineered PET residue was parametrized using the RED server with QM optimization using Gaussian.^27^

### MD Simulations

The CHARMM-GUI web server was then used to create hydrated, membrane-bound MCR-1 systems for MD simulations.^28^ The protein was oriented in the membrane using the PPM server^29^ option on the protein chain. A heterogeneous lipid bilayer was built in a rectangular box, with the Z dimension determined by the thickness of the water shell (20 Å) and the *X* and *Y* dimensions by the numbers of lipid components, keeping the two values equal to one another. The bilayer was composed of a mixture of palmitoyloleoyl phosphoethanolamine (POPE) and palmitoyloleoylphosphatidylglycerol (POPG) lipids in an approximate 80:20 ratio. The CHARMM-GUI Membrane Builder tool^30^ was used to automate the process of embedding the protein into the lipid bilayer. The tool calculates the number of lipids based on the requested membrane dimensions and composition using experimentally derived area-per-lipid values. Overlapping lipids are replaced, and the remaining lipids are redistributed to ensure symmetry and a balanced pressure field. The upper leaflet was composed of 302 POPE and 75 POPG lipids, and the lower leaflet was composed of 320 POPE and 80 POPG lipids, to account for the reduced cross-sectional area of the protein on the intracellular (lower) side relative to the extracellular (upper) side of the membrane. This produced a final system size of *c*. 156 × 156 × 124 Å. The membrane was built using a replacement method, which involved one POPE residue being modeled into the MCR-1 active site (see below). CaCl_2_ ions were included using a distance ion-placing method^30^ to neutralize the system, with a final concentration of 0.1 M. Input files were generated by the web server for AMBER using the NPT ensemble at 300 K, with ff14SB^31^ for protein, lipid17 ^32^ for lipids, and TIP3P for water^33^ after membrane building.^30,34^ The bound zinc ion(s) were parameterized using the standard 12–6 Lennard-Jones (LJ) nonbonded model.^35^

During the membrane-building step, a POPE phospholipid was modeled into the active site, with the position of one of the two acyl tails based on the position of the acyl chain of the dodecyl-beta-d-maltoside (DDM) detergent molecule in the EptA structure (pdb 5FGN), keeping the PEtN headgroup in the plane of the membrane surface. This POPE molecule was deleted from the system in models without the bound phospholipid. The various systems were minimized using the AMBER engine pmemd.MPI for 500 steps using a nonbonded cutoff of 9.0 Å, with harmonic positional restraints on the protein (weight 10.0) and on the membrane (weight 2.5). Long-range electrostatic interactions were treated by the particle mesh Ewald (PME) method. NPT equilibration was performed at 300 K (except where stated) using the Berendsen barostat and Langevin dynamics with γ = 1.0 ps^–1^, a nonbonded cutoff of 9.0 Å, and applying SHAKE^36^ restraints. The equilibration was performed over six steps, with parameters specified in Table S1. For each system tested (mono- and dizinc MCR-1, each simulated without and with Thr285 modified by covalent addition of phosphoethanolamine, and with and without bound POPE), three production runs, each of 500 ns, were then carried out.

A further set of simulations was carried out for the four systems containing dizinc MCR-1, with restraints applied to zinc co-ordination. Where employed, coordination of the Zn1 (by Glu246 OE2, Thr285 OD1, Asp465 OD1, and His466 NE2) and Zn2 (by His395 NE2 and His478 NE2) zinc ions was restrained to the distances observed in the crystal structure 5LRM,^16^ and the pairwise angles between them (set to reference value ± 10.0°) with restraint values of 25 kcal mol^–1^ Å^–2^.

Principal component analysis (PCA) of trajectories was carried out using the MDAnalysis PCA tool.^37^ Gaussian mixture analyses of interdomain distances and angles (defined as the angle between the center-of-mass (COM) of the catalytic domain, the COM of the zinc-binding residues Glu246, Asp465, and His466, and the COM of the transmembrane domain) were carried out using the sklearn.mixture tool within scikit-learn^38^ to analyze measured distances/angles for every frame (once per ns) over 50–500 ns for each replicate simulation. Measurements of membrane thickness across the area simulated, and of the area-per-lipid in upper and lower membrane leaflets, were performed using GRIDMATMD.^39^ The membrane was divided into a 40 × 40 grid, resulting in individual cells ∼0.39 nm across. The phosphate atoms of the lipid headgroup were used as reference points for lipid positions at each time frame. Measurements used snapshots taken every 20 ns from 50 to 500 ns of triplicate simulations for each system.

### Colistin Susceptibility Testing

The complete MCR-1 open reading frame was amplified from previously generated constructs^16^ by PCR using KOD DNA hot start polymerase (Sigma-Aldrich) and primers specified in Table S2. Purified products were subsequently recombined with pBAD*/Myc*-HisC (Thermo Fisher), linearized with restriction enzymes Nco1 and SnaB1 (New England Biolabs), using the In-Fusion HD cloning kit (Takara Bio) according to manufacturer’s instructions. Recombination reactions were transformed into Stellar (Takara Bio), and plasmids were recovered from recombinant colonies and sequenced (Eurofins Genomics) to confirm the integrity of the recombinant construct. MCR-1 Glu116Ala and Glu116Leu mutants were then generated using the QuikChange II XL Site-Directed Mutagenesis Kit (Agilent Genomics) according to manufacturer’s instructions, with reaction cycles increased to 25 and primers as specified in Table S2. Mutagenesis reactions were transformed into XL10 Gold (Agilent Genomics), and plasmids were recovered from recombinant colonies and sequenced (Eurofins Genomics) to confirm the presence of the desired mutation and integrity of the remaining sequences.

pBAD*/Myc*-HisC plasmids containing wild-type and mutant MCR-1 were transformed into DH5α^40^ for evaluation of protein expression and measurement of colistin minimal inhibitory concentration (MIC). MICs were measured by broth microdilution according to European Committee on Antimicrobial Susceptibility Testing (EUCAST) guidelines, in biological triplicate, in cation-adjusted Mueller–Hinton medium (Becton Dickinson) supplemented with 50 μg/mL carbenicillin to maintain plasmid selection and arabinose as specified to induce MCR-1 expression. Briefly, bacterial cells from fresh agar plates were resuspended to an optical density (OD) at 600 nm corresponding to 0.5 McFarland standard (approximately 1.5 × 10^8^ colony-forming units (cfu)/mL), and the resulting suspensions were used to inoculate 96-well microtiter plates at colistin concentrations ranging from 0.25 to 128 μg/mL. Plates were incubated overnight at 37 °C for 18 h, and optical density was read at 600 nm on a Clariostar plate reader (BMG Labtech).

Expression of wild-type and mutant MCR-1 in recombinant DH5α was assessed by Western blotting using an anti-*Myc* antibody. Briefly, cells from overnight cultures grown at 37 °C with shaking in lysogeny broth (LB), supplemented with 50 μg/mL carbenicillin and arabinose as specified, were recovered by centrifugation and resuspended in 50 mM HEPES pH 7.5, 400 mM NaCl, and 0.1 mM ZnCl_2_ to an OD at 600 nm of 2.5. 100 μL of each sample was added to 100 μL of SDS loading dye (New England Biolabs) containing β-mercaptoethanol; 20 μL was loaded onto a precast SDS-PAGE^41^ gel (NuSep), alongside a prestained PageRuler protein ladder (ThermoFisher); and the gel was run for 50 min at 170 V before transfer onto a nitrocellulose membrane (Bio-Rad blot transfer pack, Transblot Turbo program, 7 min, 25 V). Membranes were blocked for 1–2 h in 20 mL of phosphate-buffered saline (PBS) with 0.05% Tween-20 (Sigma) and 1.25 g of skimmed milk powder, and then stained for 1–2 h with primary (GeneTex GT0002 anti-*Myc* 1:5 000 dilution) and secondary (Sigma-Aldrich rabbit antimouse IgG, horseradish peroxidase-conjugated; 1:10 000 dilution) antibodies. Immobilized antibodies were stained with the Metal Enhanced DAB Substrate Kit (ThermoFisher) and imaged using a G:BOX Chemi XX6 instrument (Syngene).

## Results

### Dynamic Behavior of Full-Length MCR-1

In previous work,^16,22,42^ we applied structural, microbiological, and computational methods to investigate the mechanism of the MCR-1 phosphoethanolamine transferase, focusing on the isolated periplasmic catalytic domain as a tractable model system. Here, we extend these studies to address the behavior of the intact (full-length) enzyme, including both the catalytic and N-terminal transmembrane (TMD) domains, in a representative inner membrane system, focusing on the interactions between the two domains and the full-length protein with the zinc cofactor and palmitoyloleoyl phosphoethanolamine (POPE) donor substrate. In the absence of an experimental structure of full-length MCR-1, these investigations utilized a homology model (Figure S1) constructed based on our experimental structure of the MCR-1 catalytic domain (PDB 5LRN^16^) and the crystal structure of the homologous EptA enzyme (PDB 5FGN^15^). The model comprises MCR-1 residues 11–541, with the first ten residues, which form an unstructured N-terminal tail, being excluded due to the absence of any predicted secondary structure, structural, or catalytic function, as well as their situation far from the active site. The model was then tested in triplicate 500 ns molecular dynamics (MD) simulations in a rectangular model membrane system comprising a mixture of POPE and palmitoyloleoylphosphatidylglycerol (POPG) in an 80:20 ratio. Eight sets of simulations tested MCR-1 in the mono- and dizinc forms, each in the presence and absence of a molecule of POPE docked into the active site cavity and with and without covalent attachment of phosphoethanolamine (PEtN) to the catalytic threonine residue Thr285. Simulations involving the PEtN-bound forms represent possible states of the intermediate, expected to be formed after the transfer of PEtN from a POPE donor to MCR-1, prior to reaction with the lipid A PEtN acceptor.

MD simulations of all eight systems converged after approximately 50 ns of simulation time, in each case to structures that remained close (≤4.0 Å) to the starting model, as evidenced by plots of Cα RMSD values averaged across the latter part (50–500 ns) of the simulations (Figure S2). (Figures S3–S8 show plots of the time dependence of Cα RMSD values for the full-length protein and the catalytic and transmembrane domains; Figure S9 shows principal component analyses (PCA) of Cα positions across the complete simulation trajectories; Figure S10 provides analysis of membrane thickness, and Table S3 details the area-per-lipid for the upper and lower membrane leaflets). Comparisons of Cα RMSD values for the two individual domains identified that the major contribution is due to changes in the transmembrane, rather than the catalytic, domain. Consistent with our previous finding that the isolated catalytic domain behaves as a rigid, stable entity, comparison of catalytic domain structures in frames extracted from simulations of the truncated and full-length proteins (Figure S11) identifies only minor differences localized to three regions: residues 219–234 that form the N-terminus of the isolated catalytic domain; residues 414–424 that form a surface loop that is undefined in some crystal structures (e.g., 5LRM^16^) and sits close to the membrane surface; and residues 298–305 that are also positioned to contact the membrane. An overall Cα RMSD of 1.79 Å, however, supports our previous conclusion that the secondary and tertiary structures of the periplasmic MCR-1 catalytic domain are largely unaffected by the presence of the transmembrane domain and/or the membrane itself.

It is also evident that the presence of the POPE donor substrate does not exert any significant effect on the overall structure of either domain. The largest differences in Cα RMSD values between equivalent sets of simulations in the absence and presence of POPE are observed for the “resting” (i.e., with Thr285 unmodified) state of the catalytic domain and for the transmembrane domain in the PEtN-bound intermediate state, both in the monozinc form. In both cases, we consider these differences to arise from stochastic variations between individual simulations in each set of triplicate repeats. For the unmodified monozinc system, this variation is due to a reorientation of the two domains relative to one another in one of the three POPE-bound simulations, as shown in Figure 2A. This is associated with an increase in the z-component of the distance between the centers-of-mass (COM) of the catalytic domain and the membrane (see below) from 43.9 Å to 49.4 Å, and an increase in the angle between the two domains (defined below) from 158.0° to 163.5°. In the intermediate (PEtN-bound) system, the major differences involve unstructured loop regions, far from the active site, in both catalytic and transmembrane domains. Less pronounced (≤4.0 Å) differences are also evident in all four helices of the transmembrane domain, which interact with the POPE molecule (below). Overall, we conclude that increases in fluctuations within the MCR-1 structure in the presence or absence of docked POPE are due to stochastic variations between repeat simulations and/or minor rearrangements within the transmembrane domain local to the POPE molecule and do not indicate long-distance or larger-scale conformational changes.

**Figure 2:**
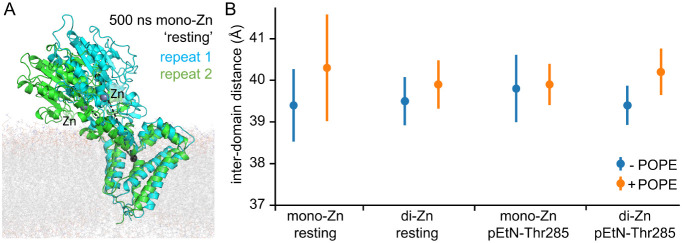
Movement of the Catalytic Domain Relative to the Transmembrane Domain in MD Simulations of Membrane-Bound MCR-1. (A) Comparison of domain orientations in the final frames of two 500 ns MD simulations (repeat 1, cyan; repeat 2, green) of monozinc MCR-1 with a POPE molecule docked in the lower active site cleft. Repeat 2 closely matches the starting structure (C_α_ RMSD 2.98 Å ± 0.15). Centers of mass (used for calculations in panel (b)) for the transmembrane (residues 11–218) and catalytic (residues 219–541) domains are shown as black spheres; dashed lines show the distance between the two. Zinc ions are shown as gray spheres. (B) Average interdomain distances during MD simulations. Distances between domain centers of mass in each of the eight sets of simulations are calculated as the means of all distances (one measurement per ns between 50 and 500 ns of each simulation) for the three replicate simulations of each system, plotted as circles ±2 SD.

### Domain Orientations During Simulations of Membrane-Bound MCR-1

These analyses indicate that the complete MCR-1-bound system retains a conformation close to that of the starting structure throughout the duration of the simulations; i.e., that both the structures of the individual domains and their orientations relative to one another are largely preserved during 500 ns of MD simulations in this model membrane environment. To further verify the latter conclusion, the distances between the respective centers of mass of the catalytic domain (residues 219–541) and the transmembrane domain (residues 11–218) were plotted over time (50–500 ns) as averages ±2 standard deviations (Figure 2B). Consistent with analysis of Cα RMSD values, the interdomain distance remains within a constant range throughout the simulations, providing no evidence that, over a time scale of 500 ns, changes in zinc stoichiometry, PEtN addition to Thr285, or presence or absence of the POPE donor substrate result in global changes to MCR-1 conformation. Specifically, none of our simulations identify the substantial changes in interdomain distance that would be expected if MCR-1 were undergoing conformational transitions involving large-scale motions of the catalytic domain relative to the transmembrane domain, as previously observed in EptA.^15^ As shown in Figure 2A, while some variation in domain positions in the individual simulations is evident—reflecting both repositioning of the transmembrane domain within the membrane and some limited reorientation of the two domains relative to one another—we do not observe the loss of interdomain interactions, and close association of the periplasmic domain with the membrane surface, described by Anandan et al. in their studies of EptA.^15,23^

In that work, such motions were observed in simulations at both 298 and 310 K. Accordingly, to investigate whether MCR-1 might experience increased mobility in simulations at physiological (i.e., host) temperature, a further set of triplicate 500 ns simulations of MCR-1 in the monozinc form, without bound POPE, was undertaken (see Figures S12–S14 for RMSD plots). The results failed to identify any significant change in dynamic behavior in simulations at the two different temperatures, as evidenced by averaged Cα RMSDs for simulations of the complete protein (2.70 ± 0.29 Å, 3.43 ± 0.48 Å); catalytic (1.55 ± 0.17 Å, 1.60 ± 0.16 Å); and transmembrane domains (2.73 ± 0.35 Å, 3.08 ± 0.48 Å) at 300 and 310 K, respectively. Similarly, no significant difference was evident in the interdomain distance (39.4 ± 0.87 Å at 300 K, and 40.1 ± 0.57 Å at 310 K).

As a further investigation of interactions between the domains of MCR-1, and between the periplasmic catalytic domain and the membrane, we also monitored the z-component of the distance between the centers-of-mass (COM) of the catalytic domain and the membrane, as well as the interdomain angle (defined as the angle between the COM of the catalytic domain, the COM of the zinc-binding residues Glu246, Asp465, and His466, and the COM of the transmembrane domain) over the course of our simulations. The distributions of both distances and angles across the duration of the simulations could be described by a bimodal Gaussian mixture model, indicating that the different systems are able to sample alternative conformations. However, the narrow distributions of both distances and angles (Figure S15) do not provide evidence that this sampling involves the extensive reorientation of the two domains necessary to sample the conformation described for EptA.

### Interactions of Full-Length MCR-1 with Zinc Ions

Multiple crystal structures have been determined for the isolated soluble periplasmic catalytic domain of MCR-1.^16,17,19,20,43^ These include a range of metalation states (i.e., zinc equivalents) and the PEtN acceptor residue Thr285 in both unmodified and phosphorylated forms. On the basis of these structures, we have used MD simulations and QM cluster model calculations^22,42^ to propose that, in the isolated catalytic domain, the Zn1 ion is stably coordinated, while the Zn2 site can retain its metal ion only in the presence of the lipid A acceptor, i.e., during the final step of the PEtN transfer reaction from Thr285 to the lipid A phosphate. However, such simulations necessarily neglect the possible involvement of residues from the transmembrane domain in zinc coordination. Accordingly, we used our MD simulations to study the stability and coordination of both zinc ions in full-length MCR-1, in the absence and presence of PEtN bound to Thr285.

As in our previous studies of the isolated catalytic domain,^42^ in monozinc MCR-1, the zinc ion remains stably bound in the Zn1 site for the full 500 ns of our simulations. As before, in these simulations, Zn1 favored six-coordinate geometry as a consequence of our use of the Lennard-Jones 12–6 parameters to treat the metal ions, with this change in coordination achievable through a move to bidentate coordination by the two carboxylate ligands, Glu246 and Asp465. The main change in active site geometry observed in these simulations is a reorientation of the transmembrane domain residue Glu116 in 2 out of 3 replicate simulations, which facilitates H-bonding to the side chain of His395 (Figure S16A). Similarly, when Thr285 is modified by addition of PEtN, while Zn1 coordination now involves one of the phosphoryl oxygen atoms in place of the Thr hydroxyl group, the metal center is otherwise stable, with reorganization of the system over the time scale of the simulation involving the PEtN moiety sampling alternative orientations through fluctuating H-bonds to His395, His478, and Glu116 (Figure S16B).

In simulations of the isolated MCR-1 catalytic domain in the dizinc form, the Zn2 site, formed in crystal structures by His395 and His478, is unstable, with bound zinc generally dissociating from unmodified (i.e., with free Thr285) enzyme over the course of 300 ns MD simulations.^42^ In contrast, in equivalent simulations with full-length MCR-1, a second zinc ion is retained over the complete duration of all three 500 ns simulations reported here. However, across the three replicate simulations, Glu116 from the transmembrane domain is the only protein ligand that retains interaction with the second zinc equivalent, with interactions involving the “gating” histidine residues, in particular His395, being lost in two of the three repeats. This behavior is replicated when Thr285 is modified through addition of PEtN; i.e., from a starting point close to the position of the Zn2 ion in the crystal structure of the dizinc catalytic domain (PDB 5LRM^16^ )­the second zinc equivalent migrates away from the MCR-1 active center toward the solvent-exposed periphery of the protein, but is prevented from dissociating altogether by persistent contact with Glu116. Thus, despite the potential for residues from the transmembrane domain of MCR-1, in particular Glu116, to contribute to a more stable, physiological Zn2 site, our simulations do not support the existence of stable dizinc forms of MCR-1 in either the “resting” ground state or after modification of Thr285 through addition of PEtN.

### Interactions of Full-Length MCR-1 with the POPE Donor Substrate

As detailed above, interactions of the PEtN donor substrate POPE were investigated with various forms of MCR-1. As a starting point for these simulations, POPE was modeled into the MCR-1 active site, with the position of one of the two acyl tails based on the position of the acyl chain of the dodecyl-β-d-maltoside (DDM) detergent molecule in the EptA structure.^15^

In the various simulations of POPE-bound MCR-1, one of the two POPE acyl tails spends most of the simulation duration packed against helix α3 of the transmembrane domain (Figure 3A, residues Tyr74 - Thr94), with frequent additional interactions with helix α4 (Ala123 - Val143). Interactions with the MCR-1 active site are, however, more variable. In the “resting” (monozinc) state, where Thr285 is unmodified by PEtN, the POPE PEtN headgroup interacts with the conserved “gating” histidine residues His395 and (less commonly) His478, which bind the second zinc equivalent (Zn2) in crystal structures of the MCR-1 catalytic domain in the dizinc form. These interactions are mediated by the terminal primary amine of the PEtN headgroup. The PEtN phosphate group can then variably contact polar groups of any of three residues—Glu116, Asn108, and Tyr97—on the periplasmic face of the transmembrane domain. In one of the three replicate simulations, however, the POPE headgroup reorients to a position where it more deeply penetrates the catalytic domain, with the phosphate group approaching the terminal hydroxyl of the PEtN acceptor Thr285. This orientation, shown in Figure 3B as a snapshot of the final frame of the 500 ns simulation, has many features of a potentially reactive conformation, with a distance of 3.7 Å between the nucleophile (Thr285 Oγ) and electrophile (POPE phosphorus) of the first (PEtN transfer) step of the lipid A modification reaction. Plots of the time dependence of this distance (Figure S17) over the full duration of the simulation demonstrate that in the relevant replicate simulation (replicate 3), the POPE P atom has approached to within 4 Å of Thr285 Oγ before 200 ns has elapsed and remains so for almost the entirety of the remainder of the simulation. These data indicate this to be a stable configuration apparently primed for PEtN transfer to Thr285.

**Figure 3:**
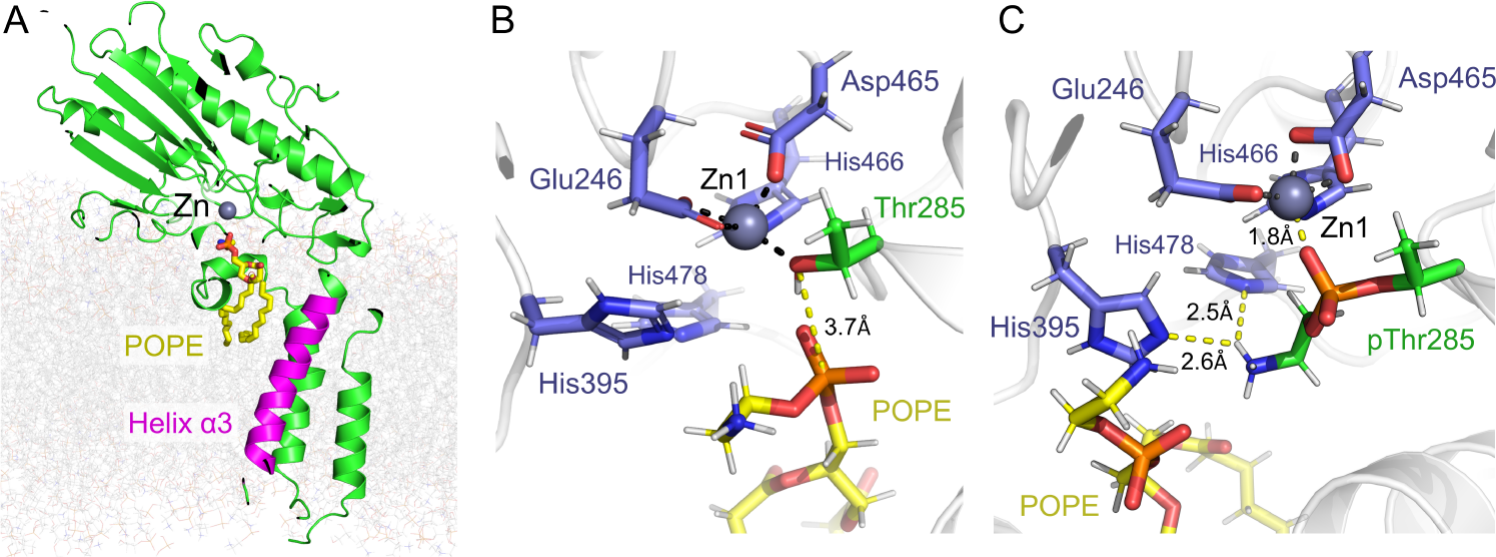
Interactions of POPE with Monozinc MCR-1. (A) Overall view of POPE (carbon atoms, yellow) bound to MCR-1, oriented in the model membrane. Zn ion is shown as a gray sphere, MCR-1 transmembrane domain helix α3 (residues 74–94) is shown in magenta. (B) Final frame from representative MD simulation of complex of monozinc MCR-1 (Thr285 unmodified) with bound POPE. POPE adopts a conformation primed for reaction with Thr285—note proximity of POPE phosphorus atom to Thr285 hydroxyl. (C) Final frame from representative MD simulation of complex of monozinc MCR-1 with phosphoethanolamine-modified Thr285 and bound POPE—note interactions of Thr285-bound phosphoethanolamine with “gating” histidine residues His395 and His478. Hydrogen and co-ordination bonds are shown as dashed lines.

Addition of PEtN to Thr285, however, prevents POPE from accessing the active site. There is a persistent interaction between the phosphate group of Thr285-bound PEtN and the active site zinc ion, replicating interactions observed in crystal structures (e.g., PDB 5LRN^16^ that contain phosphorylated Thr285, and in the simulations described above. The ethanolamine moiety can then make more transient contacts with the “gating” residues His395 and/or His478. In consequence, modification of Thr285 prevents POPE from approaching the MCR-1 zinc center (Figure 3C), leading to a loss of consistency in orientation of bound POPE in the three replicate simulations. Similarly, addition of a second zinc equivalent to the Zn2 site precludes interaction of POPE with the Zn1 site or Thr285. In these simulations, the starting complex involves Zn2 coordination by the “gating” His395 NE2, Glu116 OE1 from the transmembrane domain, and a POPE phosphoryl oxygen atom. Zn2 remains bound for the full 500 ns duration of all three replicate simulations but makes a variety of interactions as these proceed, most commonly losing contact with His395 and occupying a range of positions coordinated by combinations of POPE phosphoryl oxygens and Glu116. The situation is exacerbated by PEtN addition to Thr285, with Zn2 rapidly dissociating from its crystallographically defined binding site but remaining bound by combinations of the POPE phosphate group, Glu116, and solvent water molecules. Thus, in simulations of dizinc MCR-1 with both free and PEtN-modified forms of Thr285, POPE remains bound via interactions of the acyl chains with the transmembrane domain, but is neither positioned nor oriented for interaction or reaction with the catalytic Zn1 center (Figure S18). Plots of the time dependence of the distance between the MCR-1 active site, defined as the center of mass of the zinc-binding residues Glu246, Asp465, and His466, and the closest POPE molecule (Figure S19) reveal that bound POPE is retained in all simulations of the various forms of MCR-1 (mono- and dizinc, unmodified, and with PEtN-bound Thr285) that start from POPE-bound states. In contrast, POPE does not approach within 10 Å of the MCR-1 active site in any of the simulations in which POPE is not already in place.

### Application of Restraints to Zinc Coordination in Simulations of Di-Zinc MCR-1

The lability of the Zn2 site is a consistent feature of the simulations described above and, notwithstanding the potential for the additional phosphoryl groups to act as zinc ligands, is not substantially reduced by PEtN addition to Thr285 or by the presence of POPE. However, both the importance to activity of the conserved potential Zn2 ligands Glu116 (see below), His395, and His478,^16^ and our previous molecular simulations suggesting that a second zinc equivalent can facilitate PEtN transfer to the lipid A acceptor,^22^ indicate a potential role for a candidate Zn2 site. Accordingly, as a further investigation of the architecture and stability of a potential Zn2 site, simulations of dizinc MCR-1 were repeated with coordinating residues to both Zn1 and Zn2 sites restrained throughout minimization, heating, equilibration, and production MD to the distances and angles measured in the crystal structure of the catalytic domain in the dizinc form (PDB 5LRM^16^). As detailed above (Materials and Methods section), restraints were then applied to interactions of Zn2 with His395 and His478, but not to those involving Glu116, as this residue is part of the transmembrane, rather than the catalytic, domain. (Plots showing the time dependence of Cα RMSD values for these simulations are presented in Figures S20–S22; Figure S23 shows principal component analyses (PCA) of Cα positions across the complete simulation trajectories; Figure S24 provides analysis of membrane thickness, and Table S3 details the area-per-lipid for the upper and lower membrane leaflets). In simulations in the absence of POPE, a stable Zn2 site, involving coordination by Glu116 as well as His395 and His478, is observed (Figure S25A). On modification by PEtN addition, the Thr285-bound phosphoryl group then “bridges” the two zinc ions, with each coordinated by an additional oxygen atom (Figure S25B). These arrangements were stable and consistent across the two sets of replicate simulations. When POPE is present, Zn2 coordination can involve both POPE and Glu116 (Figure S25C) but varies between replicates and is stabilized by PEtN addition to Thr285 to generate an arrangement whereby the Thr285 backbone rotates to enable coordination of Zn2. Consequently, the bridging interaction of the two Zn ions by the PEtN phosphoryl group is lost, and Zn2 is coordinated by the phosphoryl groups of both PEtN and POPE (Figure S25D). This stable architecture was observed in all three replicate simulations.

The distributions of the z-component distance between the centers-of-mass of the catalytic domain and the membrane, as well as the interdomain angle, were also investigated for the restrained simulations of dizinc MCR-1. These analyses (Figure S26) confirmed that the application of restraints did not substantially affect the orientations of the two domains relative to one another. Distributions of both distances and angles could be fitted to bimodal Gaussian mixture models, with the ranges of distances (c. 36–54 Å) and angles (c. 143° to 172°) closely matching those observed for the unrestrained simulations (above).

As detailed above, Zn2 coordination by Glu116 is a feature of simulations of dizinc MCR-1 in which restraints are applied to interactions involving other coordinating residues (His395 and His478), suggesting that this residue is a likely contributor to a putative Zn2 site and may then be of functional importance. Accordingly, to investigate the possible importance of Glu116 to MCR-1 activity, Glu116Leu and Glu116Ala mutants of MCR-1 were generated in the arabinose-inducible pBAD vector, and the colistin MIC values of recombinant were measured in the presence and absence of arabinose induction (Table S4). In the absence of arabinose, a colistin MIC of 2 μg/mL was measured for bacteria expressing wild-type MCR-1, but this increased to 4 μg/mL, i.e., in the resistant range according to EUCAST breakpoints, on induction by 0.02% arabinose. In contrast, MIC values for expressing Glu116 mutants remained equal to or below those of untransformed and vector-only controls (1 μg/mL) in both the absence and presence of arabinose. Western blots (Figure S27) did not identify reductions in expression levels of Glu116 mutants compared to that of wild-type MCR-1 that would explain the discrepancy. These data therefore support Glu116 as a residue important to MCR-1 activity, consistent with the observations above and the results of others’ previous investigations by directed mutagenesis^44^ or mutational scanning^45^ approaches.

## Discussion

Despite the increased attention to polymyxin antibiotic resistance that followed the discovery of MCR-1, there remains limited information describing the interactions of the complete, full-length protein, which comprises both periplasmic catalytic and inner membrane transmembrane domains, with either its zinc cofactor(s) or its lipidated donor or acceptor substrates. Here, we address this knowledge gap by applying molecular dynamics (MD) simulations to a homology model of full-length MCR-1 in a membrane environment. In previous MD simulations of the isolated periplasmic catalytic domain,^42^ based on crystal structures, we established that the overall structure is minimally affected by phosphorylation of the Thr285 acceptor (which can occur during expression/purification of the recombinant protein and has consequently been observed in experimental structures^16^) or by zinc stoichiometry, but that metal binding to the Zn2 site is unstable. Based on these observations, and on density functional theory (DFT) and *ab initio* calculations on cluster models of the MCR-1 active site,^16,22,42^ we proposed that MCR-1 acts by a “ping-pong” mechanism whereby phosphoethanolamine (PEtN) transfers from a lipidated, phosphatidylethanolamine donor to form a phosphointermediate at Thr285 in a reaction that requires a monozinc form of the enzyme. Subsequent PEtN transfer to the lipid A acceptor is then promoted by the dizinc form of the enzyme, indicating that a second zinc equivalent is recruited to the MCR active site in either the phosphointermediate form or on lipid A binding. Alongside these findings, MD simulations by others, based on the crystal structure of the full-length form of the EptA PEtN transferase, identified this enzyme as capable of accessing an “open” form in which the periplasmic domain repositions relative to the transmembrane domain and “rolls” over the surface of the membrane.^15^ We now investigate the dynamic behavior of full-length MCR-1 in MD simulations in a model membrane, simulating both mono- and di-zinc forms of the enzyme containing unmodified and phosphoethanolamine-linked forms of Thr285, in the presence and absence of the POPE donor substrate.

At a gross structural level, two conclusions are evident. First, the overall structure of the catalytic domain, and the architecture of the MCR-1 active site, appear to be little affected by the presence of the transmembrane domain, as evidenced by comparison with our previous simulations of the periplasmic domain^42^ and the consistency of overall RMSD values for simulations carried out under the eight different sets of conditions (Figures S2–S8). Second, while there is limited motion of the two domains relative to one another, as adjudged by measurements between the respective centers of mass (Figure 2) and the distributions of distances and interdomain angles (Figures S15 and S26), we do not observe the more profound rearrangements described by Anandan et al. in their simulations of EptA,^15^ in which the catalytic domain retains only minimal contact with the transmembrane domain and instead makes extensive interactions with the polar headgroups of the membrane lipids. While under the conditions of our simulations it is possible for the two domains to move relative to one another, in none of the eight combinations of donor substrate, alternative zinc stoichiometry, and Thr285 modification did we observe the ∼15 Å increase in separation of centers of mass that indicated the profound conformational change described for EptA. This is true in simulations run at both 300 and 310 K, replicating both types of conditions under which this conformational change was observed.

There are several possible explanations for this apparent discrepancy. First, the differing behaviors may indeed reflect mechanistic differences between the two proteins, although their relatively high degree of sequence identity and structural conservation may make this unlikely. Second, the choice of parameters, in particular, the force field sets, could influence the results of the two sets of simulations. Specifically, the simulations described here were carried out using AMBER (ff14SB force field for protein, lipid17 for lipids, and TIP3P for water), as opposed to GROMACS^46^ (GROMOS 54A7 ^47^ for protein, lipid parameters derived from Piggot et al.^48^ (as detailed^15^) and SPC^49^ for water) as used by Anandan et al.^15^ There are several further differences between the two sets of simulations and their setup—the current work orients the membrane using the PPM server,^29^ whereas Anandan et al. trialed two different depths for their 100% DPPE membrane before finally using a position similar to that obtained with the PPM server. That same orientation was then used for the mixed membrane system (see below).

Third, the current work utilizes a somewhat larger membrane system, extends the duration of the production runs from 100 to 500 ns, and employs an 80:20 mix of lipids (POPE:POPG) that differs from the uniform composition (100% dipalmitoyl-*sn*-glycero-3-phosphoethanolamine (DPPE)) under which EptA was observed to undergo the striking conformational change described. Notably, such motions were not observed in simulations of EptA that used membranes comprising an 80:20 mixture of 1-palmitoyl-2-palmitoleyl-*sn*-glycero-3-phosphoethanolamine (PPoPE) and 20% 1,2-dimyristoyl-*sn*-glycero-3-phospho-(1’-rac)-glycerol (DMPG), i.e., closer to the conditions reported here. Anandan et al. also provide experimental evidence for the existence of an “open” conformation of EptA based on SAXS curves acquired from recombinant protein incorporated into *n*-dodecyl-phosphocholine (DPC) micelles,^23^ while cautioning that DPC is a known destabilizer of some membrane protein systems. Of note, the “open” conformation was not observed in equivalent experiments using DDM micelles. Overall, while it appears clear that the two domains of bacterial phosphoethanolamine transferases, such as EptA or MCR-1, are able to move relative to one another, comparison of available data indicates that in both laboratory investigations and simulations, the extent of such movements is strongly dependent upon the local environment.

Our simulations also allowed for investigation of interactions of MCR-1, in both mono- and dizinc forms and in the presence and absence of PEtN-modified Thr285, with the POPE donor substrate. Notably, in all simulations starting from POPE-bound forms, POPE remained associated with MCR-1, due in large part to interactions of the lipid tail with the transmembrane domain. However, close approach of the POPE headgroup to the active center was possible only in simulations involving the “resting” form of the enzyme, i.e., the monozinc form with unmodified Thr285. Under these conditions, POPE, which initially sampled orientations in which the PEtN primary amine contacted the conserved “gating” histidine residues His395 and His478, was (in one of three replicate simulations) able to adopt a stable conformation compatible with subsequent reaction with Thr285, i.e., with the PEtN phosphorus atom approaching Oγ of the Thr285 acceptor. Importantly, this position was not reached in simulations of dizinc MCR-1, in which the POPE phosphoryl oxygen groups preferentially interacted with the Zn2 ion, or in simulations of the monozinc enzyme with Thr285 modified by covalent addition of PEtN, where interaction of the PEtN amine with His395 and/or His478 prevented further entry of POPE into the active site. Taken together with others’ experimental observations that the “resting” as-isolated state of the purified recombinant enzyme contains a single zinc equivalent,^15,50^ these data then support mechanistic proposals wherein the first stage of the PEtN transfer reaction, i.e., addition of PEtN to Thr285, involves reaction of the donor substrate (e.g., POPE) with the mono-, rather than dizinc, form of the enzyme.

The existence and physiological relevance of a second zinc-binding (Zn2) site in MCR enzymes are a matter of ongoing uncertainty. In crystal structures of the isolated catalytic domain, this site, comprising His395 and His478, is completed by a coordinating Glu residue from the second molecule of a crystallographic dimer.^16,51^ Consequently, in simulations of the isolated catalytic domain, in which this interaction is absent, the Zn2 site is unstable.^42^ In full-length MCR-1, the conserved residue Glu116, located in the MCR-1 transmembrane domain but positioned close to the catalytic center, is an alternative candidate to complete the Zn2 site. Fulfillment of this role is one explanation for the importance of Glu116 to MCR-1 activity, as demonstrated here and previously.^44,45^ However, under all conditions tested here (presence or absence of POPE and/or PEtN addition to Thr285), the Zn2 site remained unstable unless restraints were applied to retain coordination by the His395 and His478 side chains. Thus, consistent with the observations that as-isolated full-length EptA and MCR-1 contain a single zinc equivalent, our simulations do not support the existence of a stable dizinc form of MCR-1 (in the presence/absence of POPE) in either the resting or PEtN-modified (i.e., phosphointermediate) states. Nevertheless, our previous work^22^ supports involvement of a second zinc equivalent in PEtN transfer to the lipid A acceptor, implying that the second stage of the reaction involves recruitment of an additional zinc ion. The present data, which do not provide evidence for strong stabilization of the dizinc form of the enzyme by the PEtN-modified phosphointermediate, indicate that this recruitment event will likely take place at a later stage in the reaction, potentially during lipid A binding.

## Concluding Remarks

The data presented here extend our previous computational investigations of MCR-1 by considering, for the first time, the dynamic behavior of the full-length enzyme in a model membrane environment. Over 500 ns simulations of MCR-1 in mono- and di-zinc forms, in both the unmodified “resting” and phosphointermediate states, the protein is characterized by a relatively limited degree of interdomain flexibility that is consistent across the different states and is little affected by the presence of the POPE donor substrate. In simulations that include bound POPE in the starting structure, POPE remains associated throughout the duration of the trajectories, but the headgroup samples a range of orientations in the different complexes. In simulations of the “resting” unmodified monozinc enzyme, bound POPE is, however, able to access a stable conformation, with its PEtN headgroup close to the Thr285 acceptor, that we consider represents a plausible ground-state complex for the first stage of the PEtN transfer reaction, i.e., phosphointermediate formation. This supports the contention that the monozinc form of MCR-1 is able to accept PEtN from a lipidated donor such as POPE and form the phosphointermediate. The relative stability of the Zn1 site in the different complexes contrasts with the instability of the Zn2 site under all conditions trialed, though we caution that the accuracy of such simulations is limited by the well-known challenges of parametrizing transition metal ions such as zinc for molecular dynamics simulations,^52^ and the metalation state(s) of full-length MCR-1, particularly in the phosphointermediate form, warrant further exploration at higher (quantum mechanical, QM) levels of theory. Extending such investigations to explore the interaction of MCR-1 (or related PEtN transferases) with the lipid A acceptor substrate will then represent a logical next step in understanding the activity of this increasingly important antimicrobial resistance determinant.

## Supplementary Material



## Data Availability

Data and Software Availability: Supporting data (MD trajectories in the AMBER.dcd format, individual topology .parm7 files containing the description of each individual system, and .pdb files containing the final structure from each of the three replicate simulations for each system) are available at the University of Bristol data repository, data.bris, at 10.5523/bris.13wzo73wmtjqu2otkrs0o58yxt.

## Notes

Equilibration parameters for molecular dynamics simulations of MCR-1 (Table S1); PCR primers (Table S2); area-per-lipid measurements (Table S3); colistin MIC values for MCR-expressing (Table S4); MCR-1 homology model used in simulations (Figure S1); Cα RMSD values for unrestrained MD simulations (Figure S2); time-dependence of Cα RMSD values for unrestrained MD simulations (Figures S3–S8); PCA for unrestrained simulations of full-length MCR-1 (Figure S9); measurements of membrane thickness for unrestrained simulations of MCR-1 (Figure S10); comparison of catalytic domain in simulations in isolation and in full-length MCR-1 (Figure S11); time-dependence of Cα RMSD values for unrestrained MD simulations at 300 and 310 K (Figures S12–S14); GMM fits of distributions of interdomain distance and angle for unrestrained MD simulations (Figure S15); active site snapshots from simulations of monozinc MCR-1 (Figure S16); interaction between Thr285 and bound POPE in simulations of monozinc MCR-1 (Figure S17); active site snapshots from unrestrained simulations of dizinc MCR-1 with bound POPE (Figure S18); POPE to active site distance for MD simulations of MCR-1 (Figure S19); time-dependence of Cα RMSD values for restrained MD simulations (Figures S20–S22); PCA for restrained simulations of full-length MCR-1 (Figure S23); measurements of membrane thickness for restrained simulations of MCR-1 (Figure S24); active site snapshots from restrained simulations of dizinc MCR-1 (Figure S25); GMM fits of distributions of interdomain distance and angle for restrained MD simulations (Figure S26); and Western blot showing expression of recombinant wild-type MCR-1 and Glu116 mutants (Figure S27) (PDF)

## Abbreviations

AMBER: Assisted Model Building with Energy Refinement
AMR: antimicrobial resistance
CHARMM: Chemistry at Harvard Macromolecular Mechanics
COM: center-of-mass
DDM: dodecyl-β-d-maltoside
DMPG: 1,2-dimyristoyl-*sn*-glycero-3-phospho-(1’-rac)-glycerol
DPC: *n*-dodecyl-phosphocholine
DPPE: dipalmitoyl-*sn*-glycero-3-phosphoethanolamine
LPS: lipopolysaccharide
MCR: mobile colistin resistance
MD: molecular dynamics
PET: phosphoethanolaminated threonine
PEtN: phosphoethanolamine
POPE: palmitoyloleoyl phosphoethanolamine,
POPG: palmitoyloleoylphosphatidylglycerol
PPoPE: 1-palmitoyl-2-palmitoleyl-*sn*-glycero-3- phosphoethanolamine
QM: quantum mechanics
RMSD: root mean-squared deviation;
SAXS: small-angle X-ray scattering
