# Supplementary material for: Dynamic Behavior and Substrate Interactions of the Polymyxin Resistance Determinant MCR‑1 Investigated by Molecular Dynamics Simulations in the Membrane Environment
Source: J Chem Inf Model. 2025 Jul 22;65(15):8322–34. doi: 10.1021/acs.jcim.5c01338 (PMC12344761; doi:10.1021/acs.jcim.5c01338)
Supplement: Supplementary file 1 [file ci5c01338_si_001.pdf]

Supporting Information

Dynamic Behavior and Substrate Interactions of the Polymyxin Resistance Determinant MCR-1 Investigated by Molecular Dynamics Simulations in the Membrane Environment

Emily Lythell[a,b], Jack Badley[a], Reynier Suardíaz[a,c#,d], Catherine R. Gurr[b], Catherine L. Tooke[b,e#], Philip Hinchliffe[b], A. Sofia F. Oliveira[a], Marc W. Van der Kamp[d], James Spencer[b]*, Adrian J. Mulholland[a]*

a. Centre for Computational Chemistry, School of Chemistry, University of Bristol, Cantock's Close, Bristol BS8 1TS, U.K.

b. School of Cellular and Molecular Medicine, University of Bristol, University Walk, Bristol BS8 1TD, U.K.

c. Departamento de Química Física, Facultad de Química, Universidad Complutense, 28040 Madrid, Spain.

d. School of Biochemistry, University of Bristol, University Walk, Bristol BS8 1TD, U.K.

e. Department of Life Sciences, University of Bath, Claverton Down, Bath BA2 7AY, U.K.

[#]present address

*corresponding authors Jim.Spencer@bristol.ac.uk; Adrian.Mulholland@bristol.ac.uk

Keywords: antimicrobial resistance, molecular dynamics, polymyxin, MCR-1, phosphoethanolamine transferase

Supporting Information

Table S1. : Equilibration Parameters for MCR-1 MD Simulations

Step	Time (ps)	Time step (fs)	Protein restraint (kcal·mol^{-1}·Å^{2})	Membrane restraint (kcal·mol^{-1}·Å^{2})
1	125	1	10	2.5
2	125	1	5	2.5
3	125	1	2.5	1.0
4	500	2	1	0.5
5	500	2	0.5	0.1
6	500	2	0.1	0

Table S2. Cloning and PCR Primers

Primer	Sequence
MCR-1 pBAD forward	GAGGAATTAACCATGATGCAGCATACTTCTGTGTGGTACC
MCR-1 pBAD reverse	GAGTTTTTGTTCTACGCGGATGAATGCGGTGCG
Glu116Ala forward	CAGACCGACCAAGCCGCGACCAAGGATCTATTA
Glu116Ala reverse	TAATAGATCCTTGGTCGCGGCTTGGTCGGTCTG
Glu116Leu forward	CCCTACAGACCGACCAAGCCCTAACCAAGGATCTATTAAACGC
Glu116Leu reverse	GCGTTTAATAGATCCTTGGTTAGGGCTTGGTCGGTCTGTAGGG

Table S3. Average Area-per-Lipid Values[a] for Upper and Lower Membrane Leaflets.

System[b]	Upper Leaflet Average ± SD (Å^2)	Lower Leaflet Average ± SD (Å^2)	Difference[c] Average ± SD (Å^2)
2ZrPnR	54.3 ± 0.9	55.6 ± 0.8	1.3 ± 0.4
1ZiPn	54.8 ± 0.7	55.8 ± 0.7	1.0 ± 0.4
1ZrPEn	54.5 ± 0.8	55.6 ± 0.8	1.1 ± 0.3
1ZiPy	54.4 ± 0.7	55.5 ± 0.6	1.1 ± 0.4
2ZiPyR	54.3 ± 4.9	55.2 ± 1.9	1.5 ± 2.9
2ZrPEn	54.6 ± 0.8	55.5 ± 0.8	0.9 ± 0.3
2ZiPy	54.6 ± 0.9	55.6 ± 0.7	1.0 ± 0.3
1ZrPy	54.5 ± 0.8	55.6 ± 0.8	1.1 ± 0.4
2ZrPy	54.5 ± 0.7	55.8 ± 0.8	1.3 ± 0.3
2ZiPn	54.4 ± 0.9	55.5 ± 0.7	1.1 ± 0.4
2ZiPnR	53.9 ± 0.9	55.1 ± 0.8	1.1 ± 0.3
2ZrPyR	54.1 ± 0.8	55.5 ± 0.7	1.4 ± 0.3
1ZrPEnH	56.0 ± 0.7	56.9 ± 0.6	0.8 ± 0.3

[a]Measurements (GRIDMATMD[1]) used snapshots taken every 20 ns from 50 – 500 ns of triplicate simulations for each system.

[b]Systems simulated are labelled as follows: "2ZrPEn" = di-zinc, unmodified Thr285, POPE-unbound; "1ZrPEn" = mono-zinc, unmodified Thr285, POPE-unbound; "1ZiPn" = mono-zinc, PEtN-modified Thr285, POPE-unbound; "2ZiPn" = di-zinc, PEtN-modified Thr285, POPE-unbound; "2ZiPy" = di-zinc, PEtN-modified Thr285, POPE-bound; "1ZiPy" = mono-zinc, PEtN-modified Thr285, POPE-bound; "2ZrPy" = di-zinc, unmodified Thr285, POPE-bound; "1ZrPy" = mono-zinc, unmodified Thr285, POPE-bound; "1ZrPEnH" = mono-zinc, unmodified Thr285, POPE-unbound, 310 K; "2ZrPnR" = restrained, di-zinc, unmodified Thr285, POPE-unbound; "2ZrPyR" = restrained, di-zinc, unmodified Thr285, POPE-bound; "2ZiPnR" = restrained, di-zinc, PEtN-modified Thr285, "2ZiPyR" = restrained, di-zinc, PEtN-modified Thr285, POPE-bound.

[c]Difference represents average of the absolute difference between upper and lower membrane leaflets measured for each snapshot.

Table S4. Colistin Minimal Inhibitory Concentrations for *E. coli* DH5α Expressing MCR-1 and Glu-116 Mutants

E. coli strain	Colistin MIC (- arabinose) (μg/mL)	Colistin MIC (+ 0.02 % arabinose) (μg/mL)
DH5α (cells only)	1	1
DH5α:pBAD (empty vector)	1	1
DH5α:pBAD WT MCR-1	2	4
DH5α:pBAD Glu116Ala	1	0.5
DH5α:pBAD Glu116Leu	1	0.5

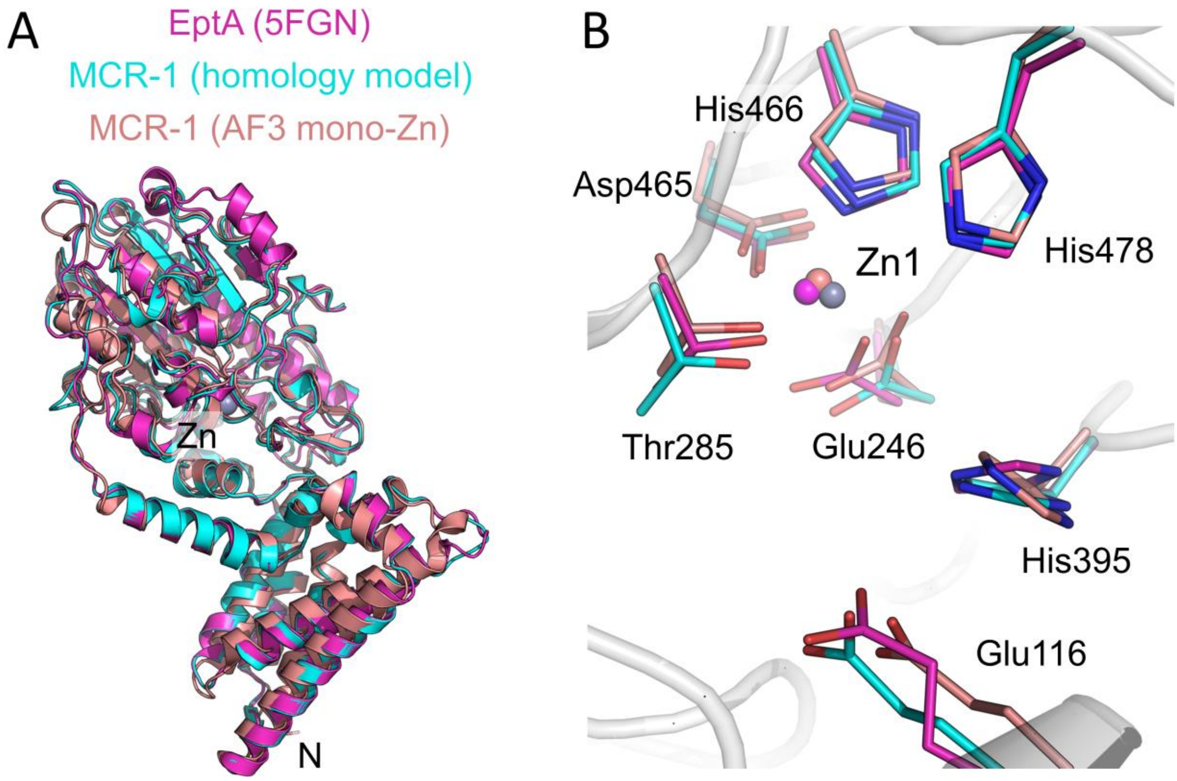

Figure S1. Comparison of MCR-1 homology and AlphaFold 3 models with *N. meningitidis* EptA. A. Homology model (cyan) of MCR-1 (full length protein, first 10 residues removed) overlaid (using PDBeFold[2]) upon the crystal structure of *Neisseria meningitidis* EptA (purple, PDB 5FGN[3]) and, for comparison, a model of MCR-1 (in the mono-zinc form) generated using AlphaFold 3[4] (brown). Calculated RMSD values are 1.28 Å over 511 Cα atoms for MCR-1 homology model versus MCR-1 Alpha Fold 3 model; 1.1 Å over 502 Cα atoms for MCR-1 homology model versus EptA; and 1.54 Å over 488 Cα atoms for MCR-1 Alpha Fold 3 model versus EptA. B. Close-ups of active sites, with zinc ion rendered as spheres and side chains of key residues shown as sticks.

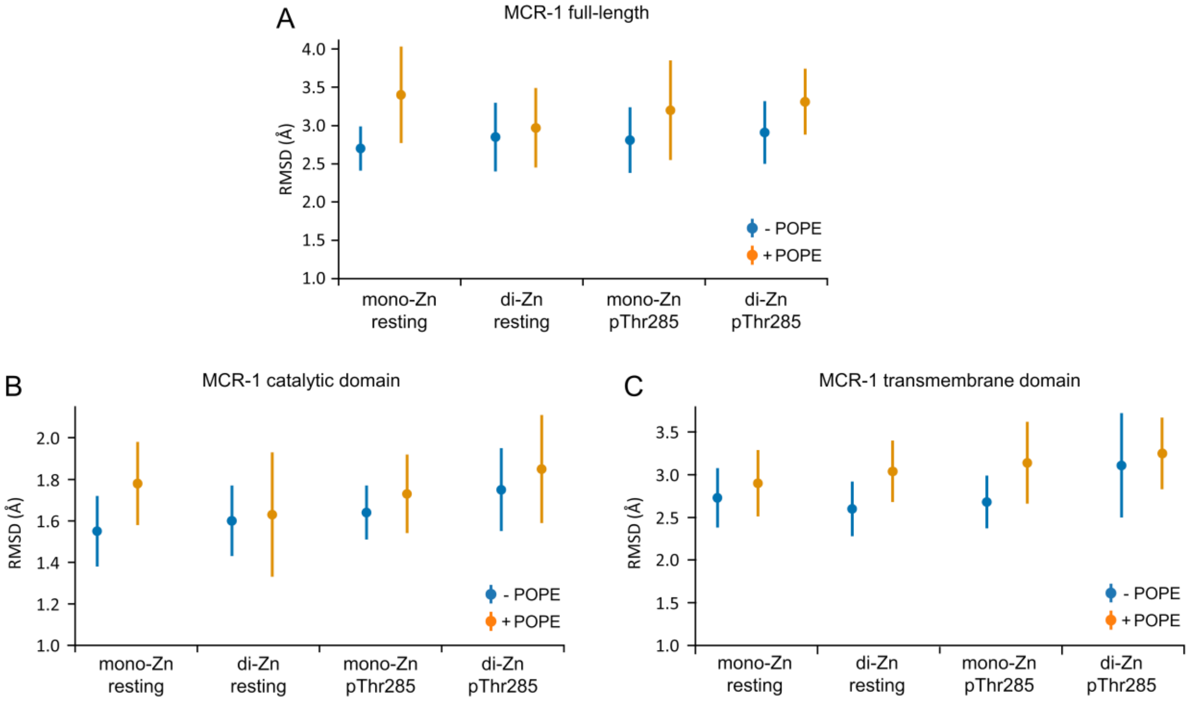

Figure S2. Average Cα RMSD Values for Unrestrained MD Simulations of MCR-1 at 300 K. Plots show mean Cα RMSD of (A) full-length MCR-1 (residues 11-541); (B) catalytic domain (residues 219-541) and (C) transmembrane domain (residues 11-218). RMSDs are averaged from 50 - 500 ns (one measurement per ns) of three replicate simulations, mean values are plotted as circles ± 2 standard deviations. "- POPE" denotes empty active site; "+ POPE" indicates simulations started with a molecule of Palmitoyl-Oleoyl Phosphatidylethanolamine (POPE) present in the active site cavity. "Resting" systems have unmodified Thr285, pThr285 systems are modeled with phosphoethanolamine covalently attached to Thr285.

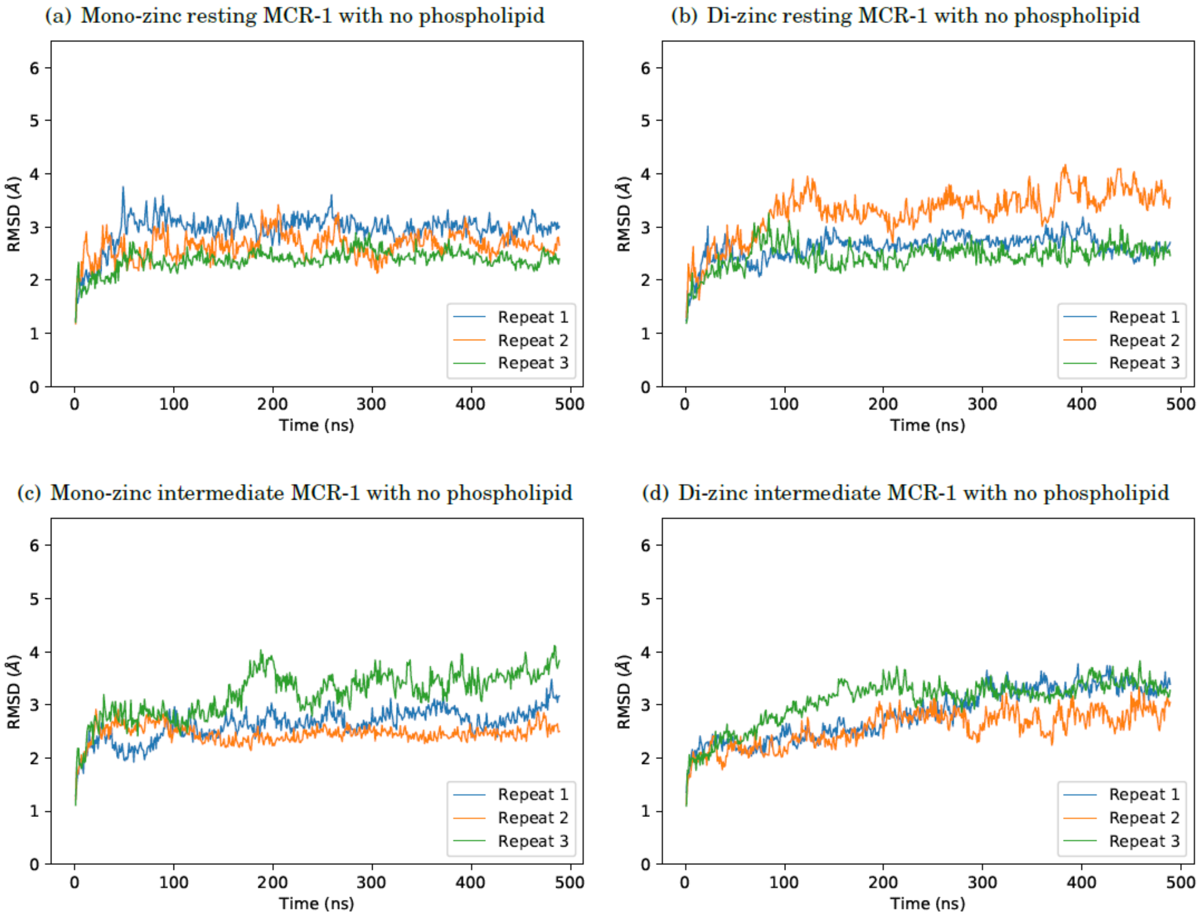

Figure S3. Time-Dependence of Cα RMSD Values for Unrestrained MD Simulations of MCR-1 at 300 K: Full-Length MCR-1 Without Bound POPE. Panels show sets of three 500 ns repeat simulations for four different systems: (a) mono-zinc MCR-1 with Thr285 unmodified; (b) di-zinc MCR-1 with Thr285 unmodified; (c) mono-zinc MCR-1 with phosphoethanolamine covalently bound to Thr285; (d) di-zinc MCR-1 with phosphoethanolamine covalently bound to Thr285. RMSD calculations were performed after aligning trajectories to the complete (residues 11 - 541) initial homology model built on EptA (5FGN[3]).

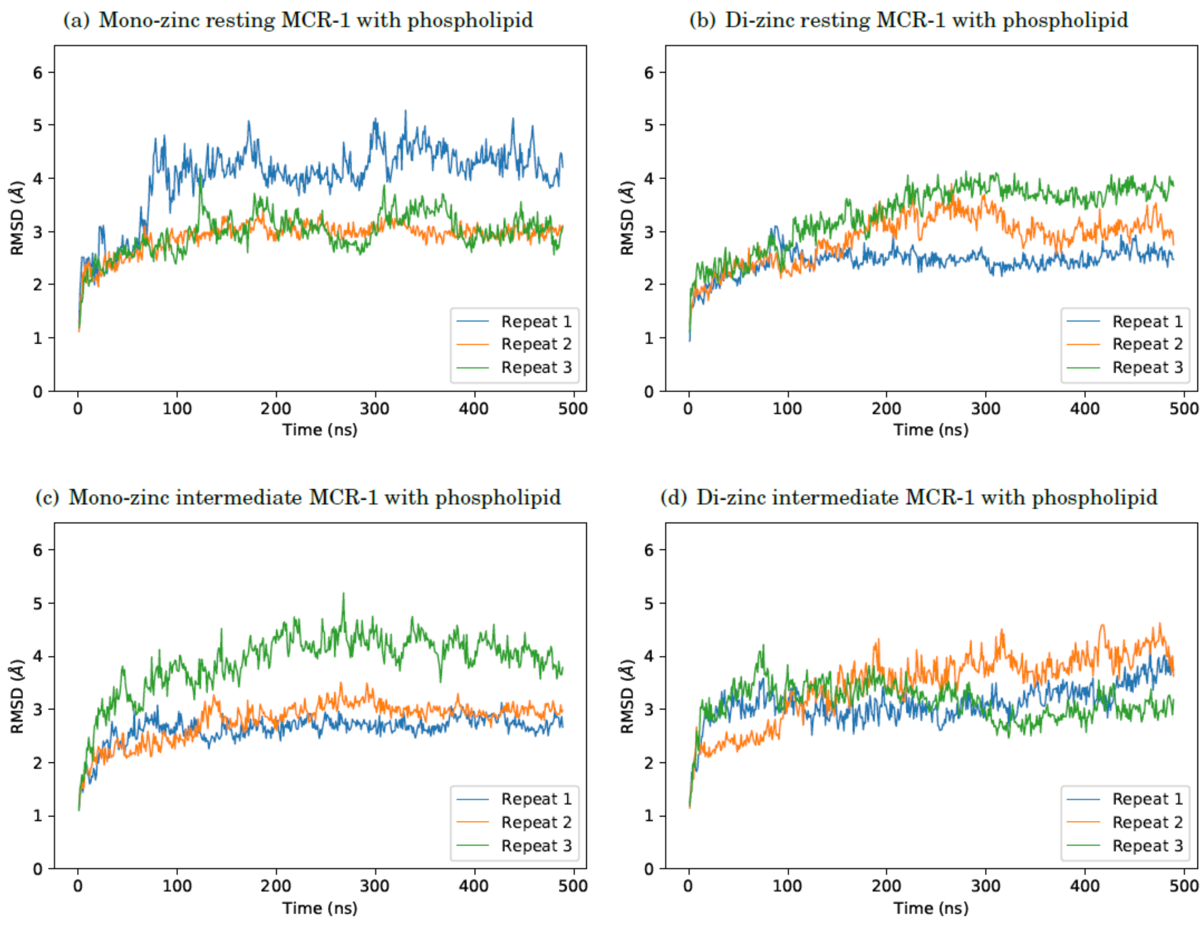

Figure S4. Time-Dependence of Cα RMSD Values for Unrestrained MD Simulations of MCR-1 at 300 K: Full-Length MCR-1 With POPE Docked Into the Active Site Cavity. Panels show sets of three 500 ns repeat simulations for four different systems: (a) mono-zinc MCR-1 with Thr285 unmodified; (b) di-zinc MCR-1 with Thr285 unmodified; (c) mono-zinc MCR-1 with phosphoethanolamine covalently bound to Thr285; (d) di-zinc MCR-1 with phosphoethanolamine covalently bound to Thr285. RMSD calculations were performed after aligning trajectories to the complete (residues 11 - 541) initial homology model built on EptA (5FGN[3]).

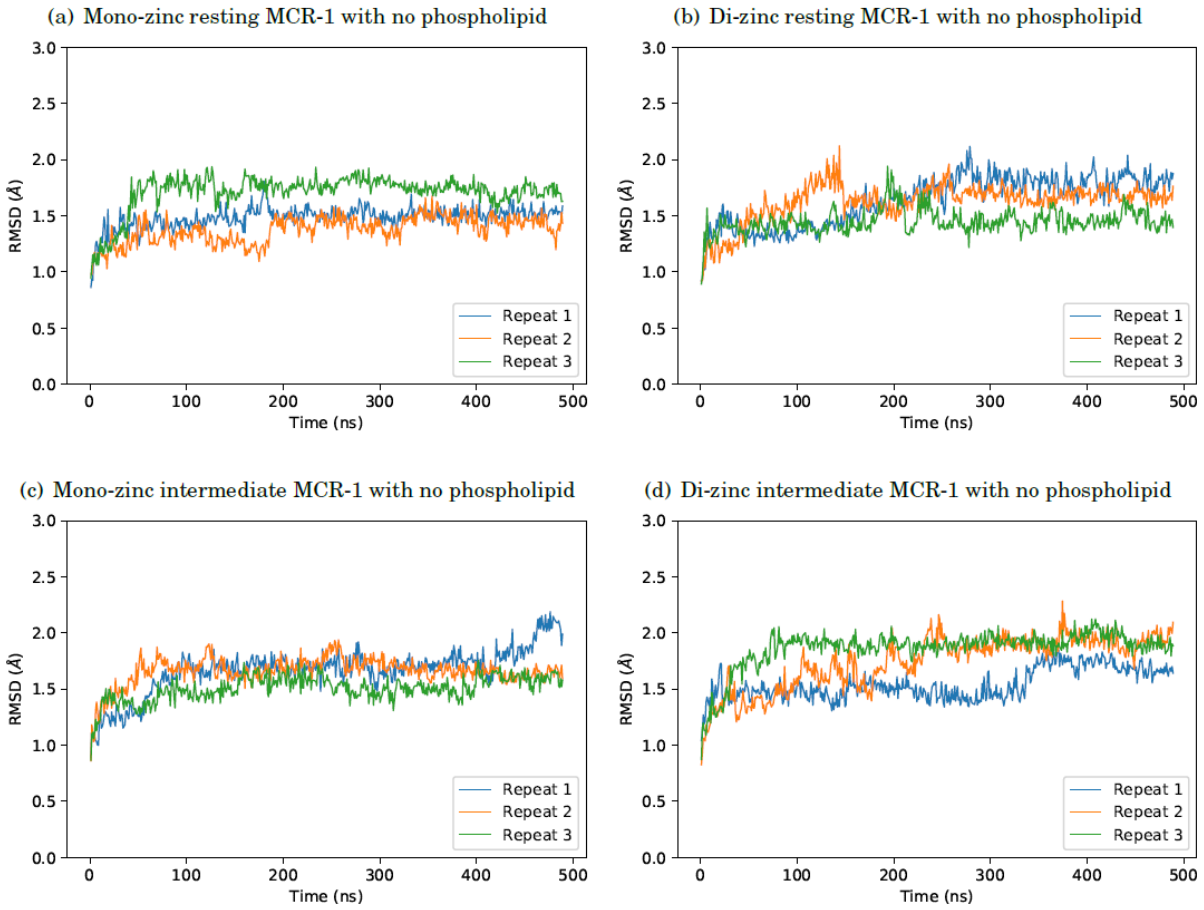

Figure S5. Time-Dependence of Cα RMSD Values for Unrestrained MD Simulations of MCR-1 at 300 K: MCR-1 Catalytic Domain Without Bound POPE. Panels show sets of three 500 ns repeat simulations for four different systems: (a) mono-zinc MCR-1 with Thr285 unmodified; (b) di-zinc MCR-1 with Thr285 unmodified; (c) mono-zinc MCR-1 with phosphoethanolamine covalently bound to Thr285; (d) di-zinc MCR-1 with phosphoethanolamine covalently bound to Thr285. RMSD calculations were performed after aligning trajectories to the catalytic domain (residues 219 - 541) of the initial homology model (Figure S1) based upon EptA (5FGN[3]).

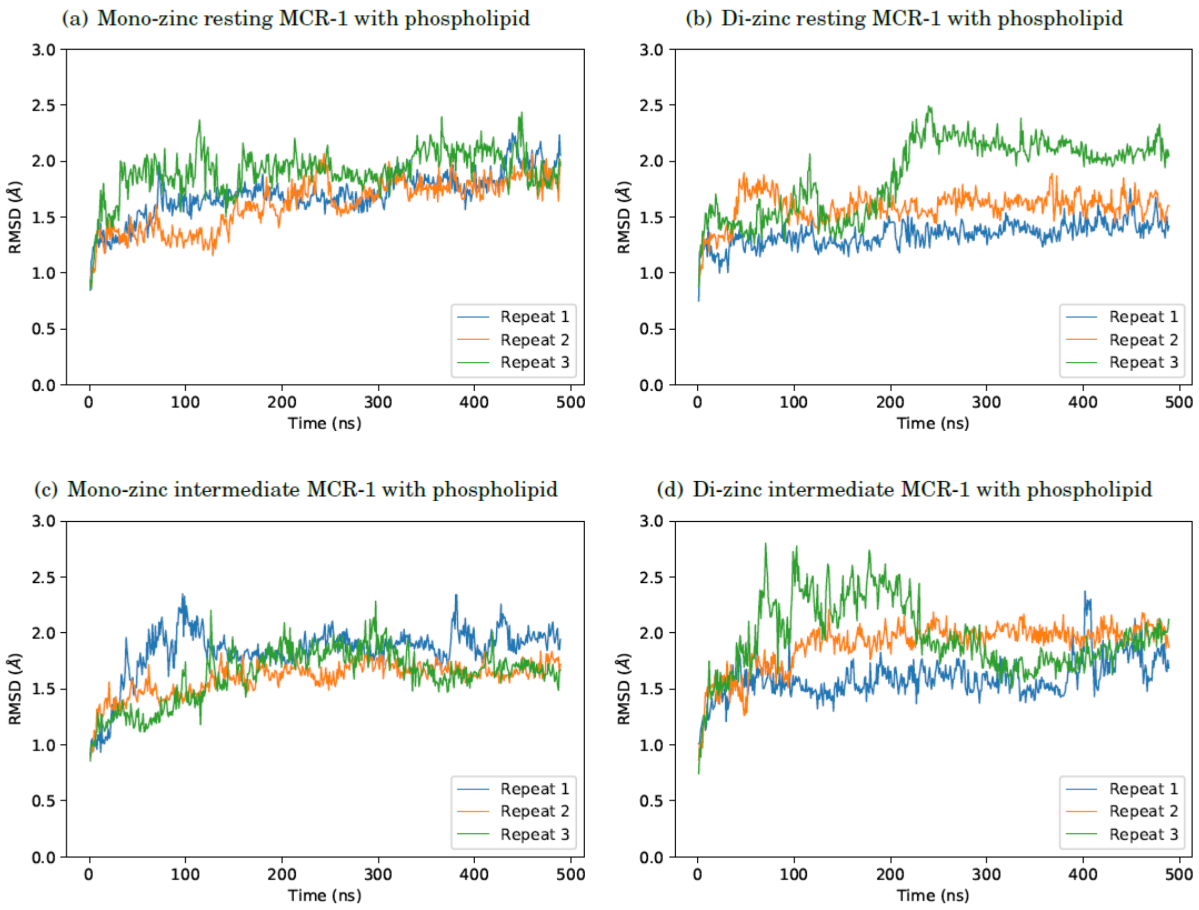

Figure S6. Time-Dependence of Cα RMSD Values for Unrestrained MD Simulations of MCR-1 at 300 K: MCR-1 Catalytic Domain With POPE Bound. Panels show sets of three 500 ns repeat simulations for four different systems: (a) mono-zinc MCR-1 with Thr285 unmodified; (b) di-zinc MCR-1 with Thr285 unmodified; (c) mono-zinc MCR-1 with phosphoethanolamine covalently bound to Thr285; (d) di-zinc MCR-1 with phosphoethanolamine covalently bound to Thr285. RMSD calculations were performed after aligning trajectories to the catalytic domain (residues 219 - 541) of the initial homology model (Figure S1) based upon EptA (5FGN[3]).

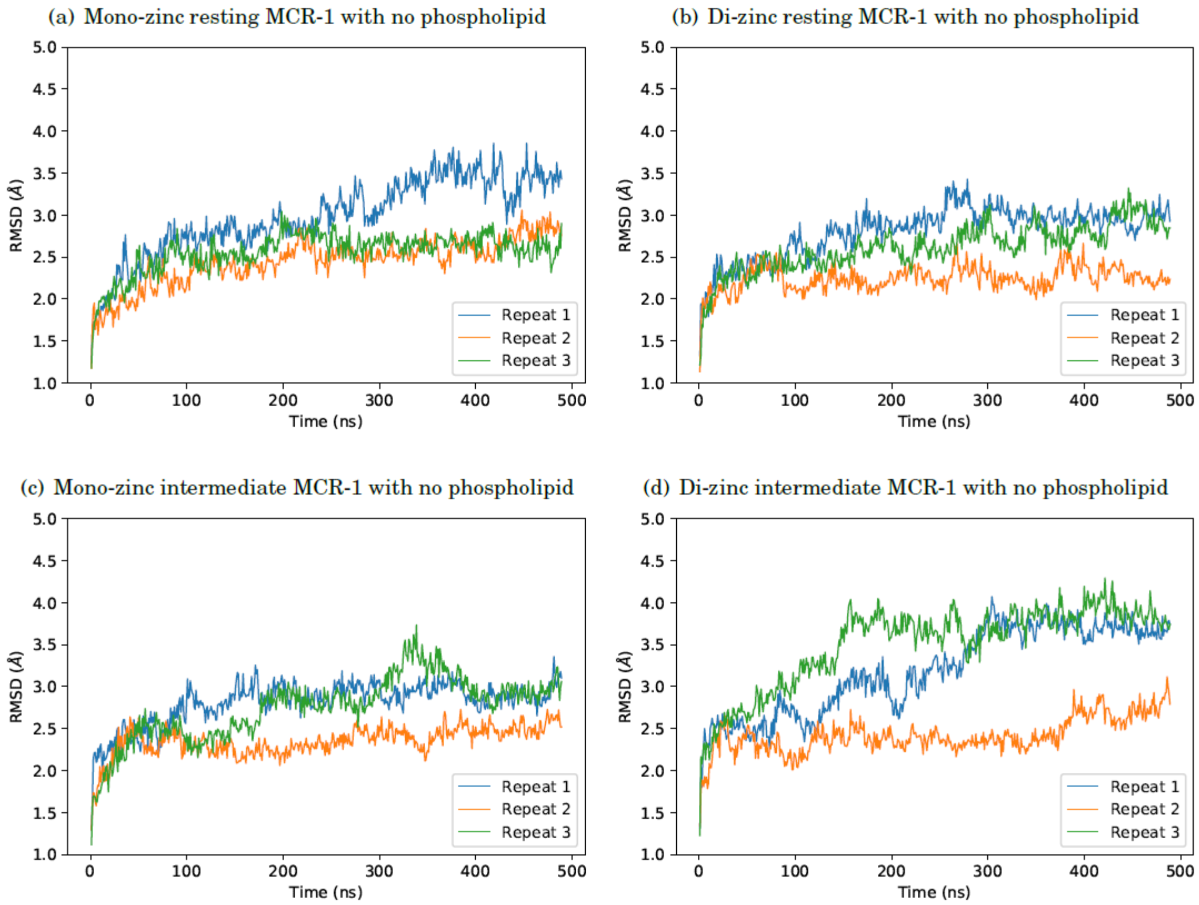

Figure S7. Time-Dependence of Cα RMSD Values for Unrestrained MD Simulations of MCR-1 at 300 K: MCR-1 Transmembrane Domain Without Bound POPE. Panels show sets of three 500 ns repeat simulations for four different systems: (a) mono-zinc MCR-1 with Thr285 unmodified; (b) di-zinc MCR-1 with Thr285 unmodified; (c) mono-zinc MCR-1 with phosphoethanolamine covalently bound to Thr285; (d) di-zinc MCR-1 with phosphoethanolamine covalently bound to Thr285. RMSD calculations were performed after aligning trajectories to the transmembrane domain (residues 11 - 218) of the initial homology model (Figure S1) based upon EptA (5FGN[3]).

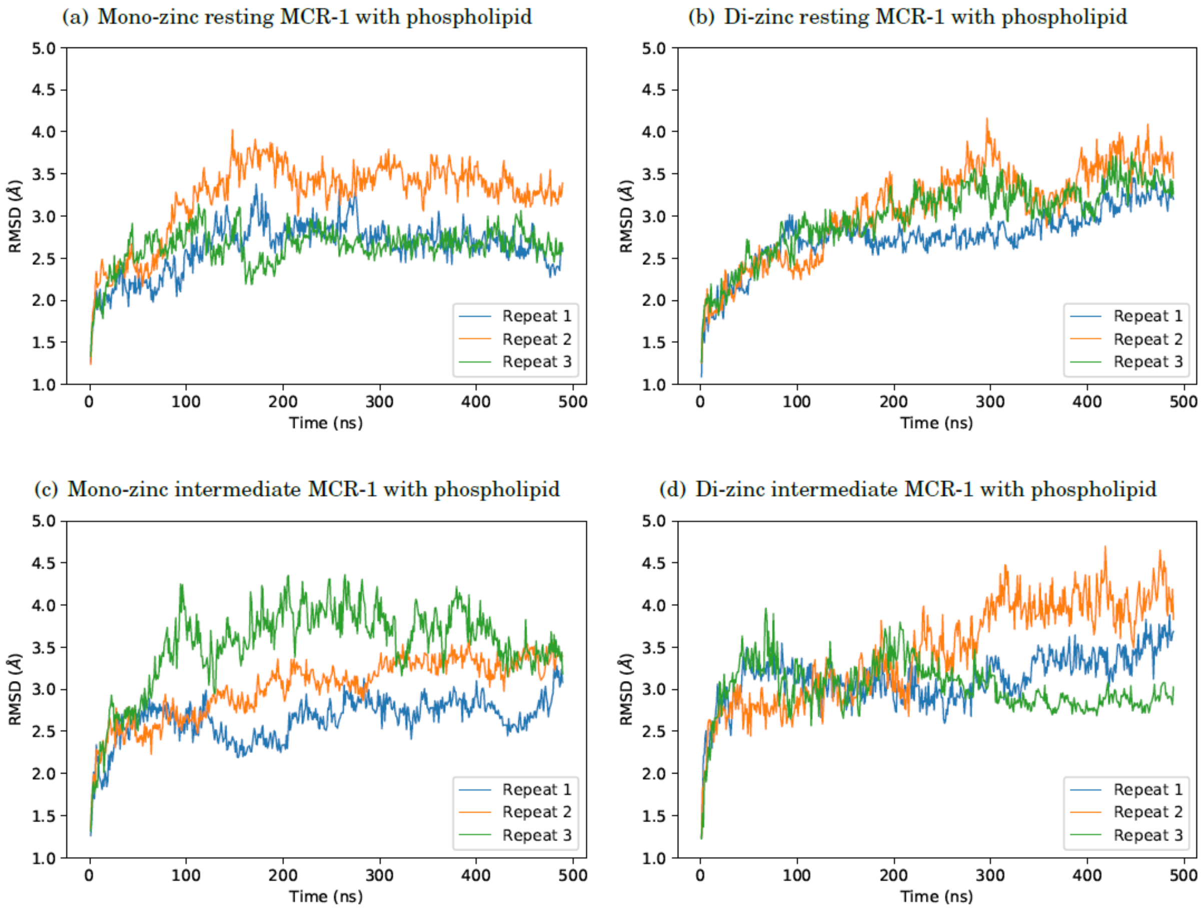

Figure S8. Time-Dependence of Cα RMSD Values for Unrestrained MD Simulations of MCR-1 at 300 K: MCR-1 Transmembrane Domain With POPE Bound. Panels show sets of three 500 ns repeat simulations for four different systems: (a) mono-zinc MCR-1 with Thr285 unmodified; (b) di-zinc MCR-1 with Thr285 unmodified; (c) mono-zinc MCR-1 with phosphoethanolamine covalently bound to THr285; (d) di-zinc MCR-1 with phosphoethanolamine covalently bound to Thr285. RMSD calculations were performed after aligning trajectories to the transmembrane domain (residues 11 - 218) of the initial homology model (Figure S1) based upon EptA (5FGN[3]).

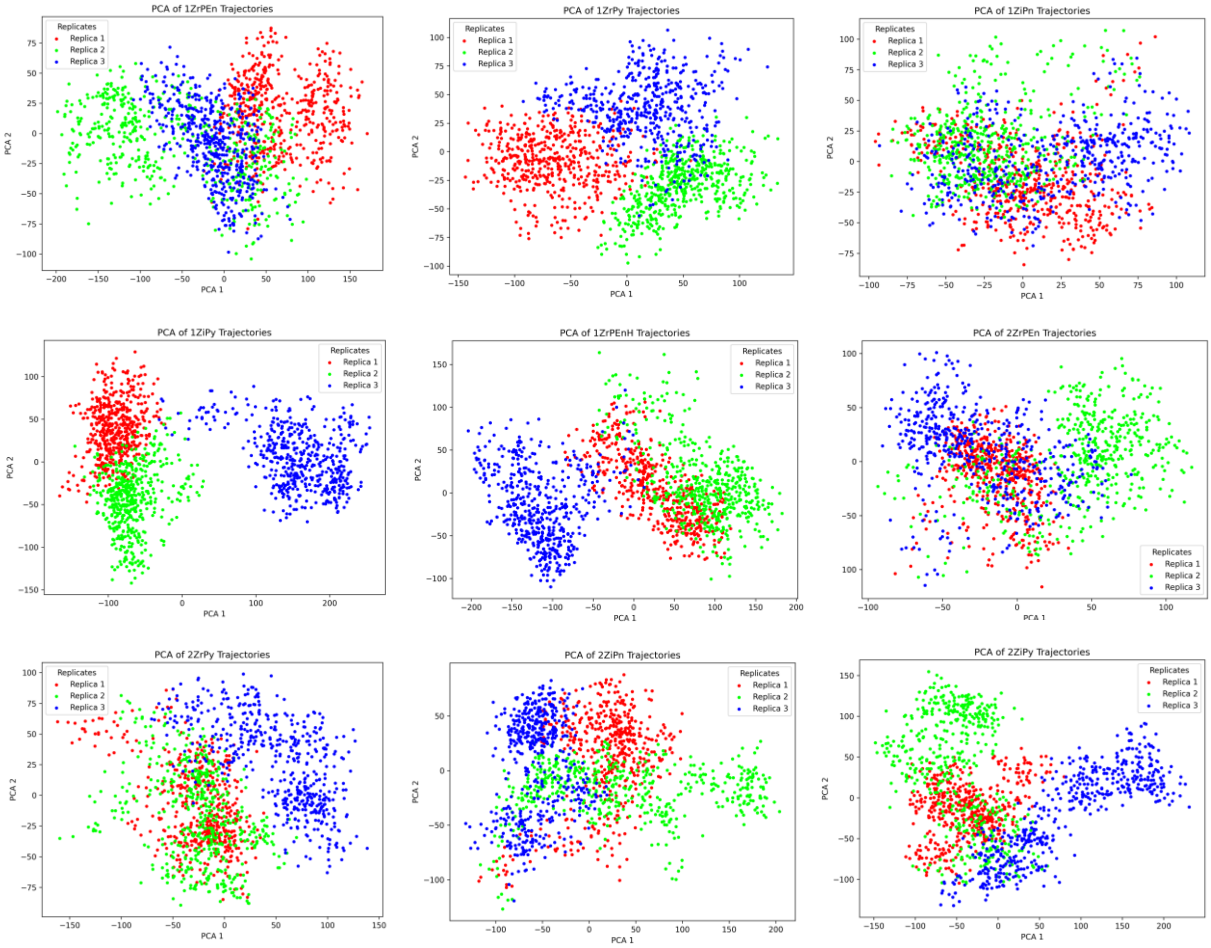

Figure S9. Principal Component Analysis (PCA) for Unrestrained Simulations of Full-length MCR-1. All three replicates for each system were combined before analysis such that they all shared a common subspace. Each trajectory was sampled once per nanosecond for the complete 500 ns of simulation (i.e. a total of 1500 frames). Analysis was carried out based on $C\alpha$ atom positions using the MDanalysis PCA tool[5]. Systems simulated are labelled as follows: “2ZrPEn” = di-zinc, unmodified Thr285, POPE-unbound; “1ZrPEn” = mono-zinc, unmodified Thr285, POPE-unbound; “1ZiPn” = mono-zinc, PEtN-modified Thr285, POPE-unbound; “2ZiPn” = di-zinc, PEtN-modified Thr285, POPE-unbound; “2ZiPy” = di-zinc, PEtN-modified Thr285, POPE-bound; “1ZiPy” = mono-zinc, PEtN-modified Thr285, POPE-bound; “2ZrPy” = di-zinc, unmodified Thr285, POPE-bound; “1ZrPy” = mono-zinc, unmodified Thr285, POPE-bound; “1ZrPEnH” = mono-zinc, unmodified Thr285, POPE-unbound, 310 K.

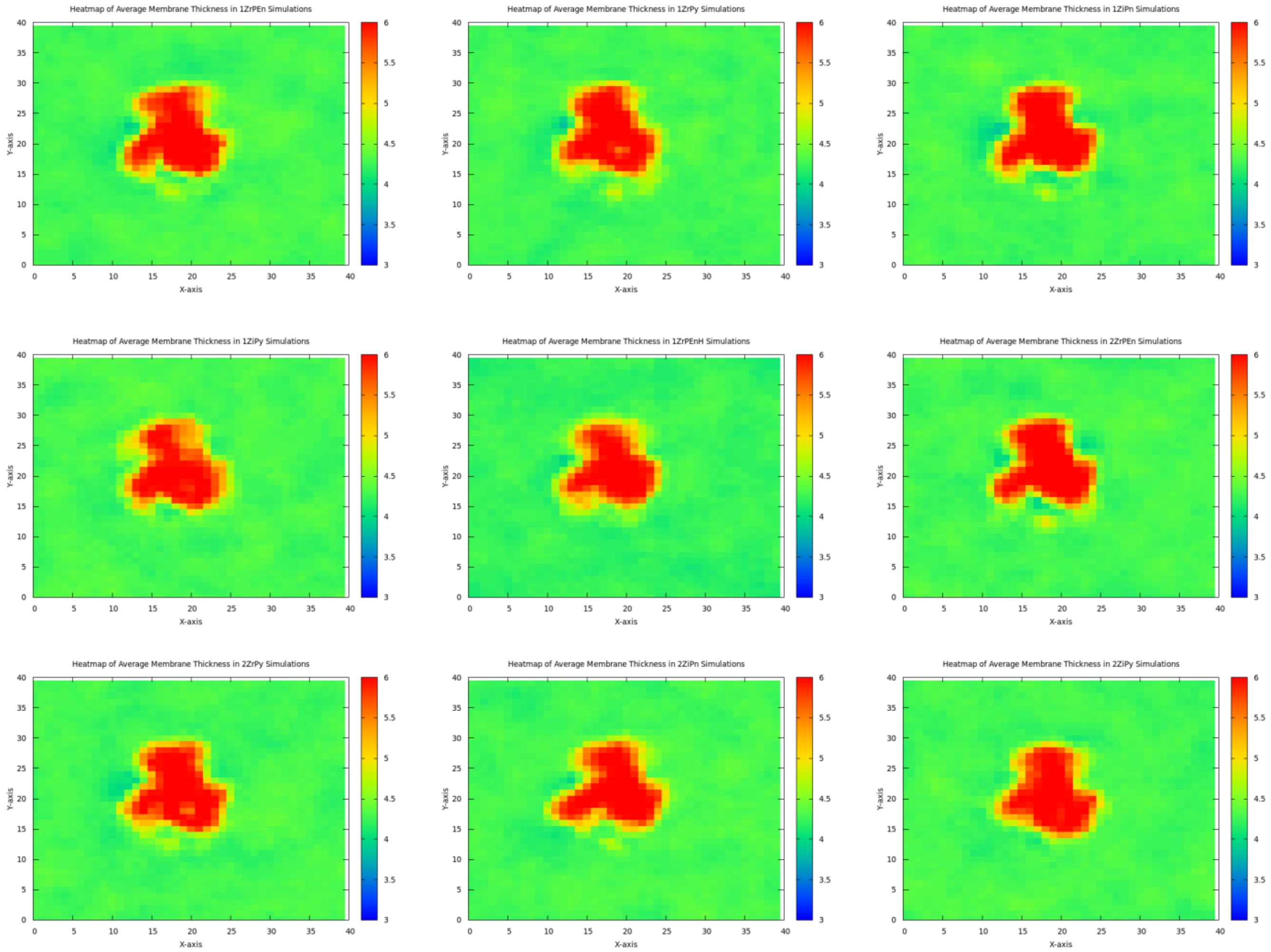

Figure S10. Measurements of Membrane Thickness for Unrestrained Simulations of MCR-1. GRIDMATMD[1] was used to measure membrane thickness. The membrane was divided into a 40 x 40 grid resulting in individual cells ~0.39 nm across. X and Y axes denote grid positions. Phosphate atoms of the lipid headgroup were used as the reference points for lipid positions at each time frame. Color bar denotes membrane thickness in nm. The height of the embedded MCR-1 protein (red) was set at 6.0 nm. Plots show average values from snapshots taken every 20 ns from 50 – 500 ns of triplicate simulations for each system. Systems simulated are labelled as follows: "2ZrPEn" = di-zinc, unmodified Thr285, POPE-unbound; "1ZrPEn" = mono-zinc, unmodified Thr285, POPE-unbound; "1ZiPn" = mono-zinc, PEtN-modified Thr285, POPE-unbound; "2ZiPn" = di-zinc, PEtN-modified Thr285, POPE-unbound; "2ZiPy" = di-zinc, PEtN-modified Thr285, POPE-bound; "1ZiPy" = mono-zinc, PEtN-modified Thr285, POPE-bound; "2ZrPy" = di-zinc, unmodified Thr285, POPE-bound; "1ZrPy" = mono-zinc, unmodified Thr285, POPE-bound; "1ZrPEnH" = mono-zinc, unmodified Thr285, POPE-unbound, 310 K.

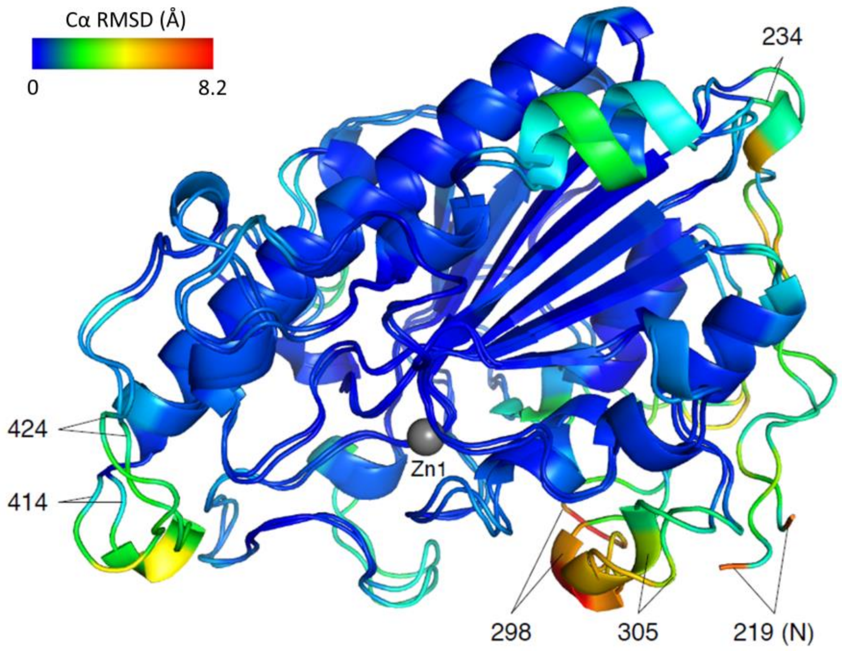

Figure S11. The MCR-1 Catalytic Domain Behaves Similarly in the Presence and Absence of the Transmembrane Domain. Two representative frames from MD simulations of the isolated MCR-1 catalytic domain[6], and the catalytic domain in full-length MCR-1 in the membrane environment (in the absence of bound POPE), both in the mono-zinc form withThr285 unmodified, were compared. Cα atoms were aligned and colored (color bar at top left) according to pairwise RMSD values (overall Cα RMSD is 1.787 Å over 323 atoms).

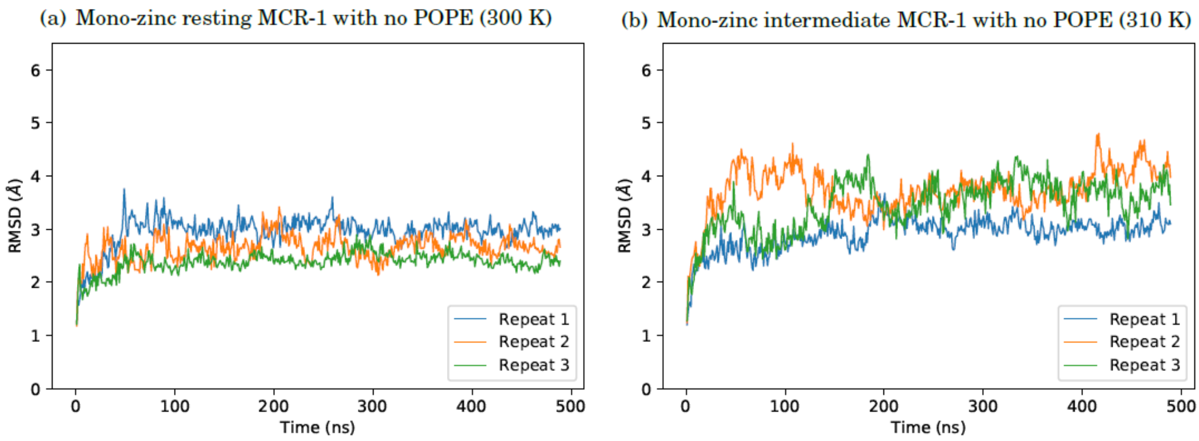

Figure S12. Time-Dependence of Cα RMSD Values for Unrestrained MD Simulations of MCR-1 at 300 and 310 K: Full-Length MCR-1. Panels show three repeat 500 ns simulations of two otherwise identical systems, run at 300 K (approximating room temperature) and 310 K (approximating body temperature). Both systems are mono-zinc full length MCR-1 with Thr285 unmodified and without bound POPE. RMSD calculations were performed after aligning trajectories to the complete (residues 11 - 541) initial homology model (Figure S1) built upon EptA (5FGN[3]).

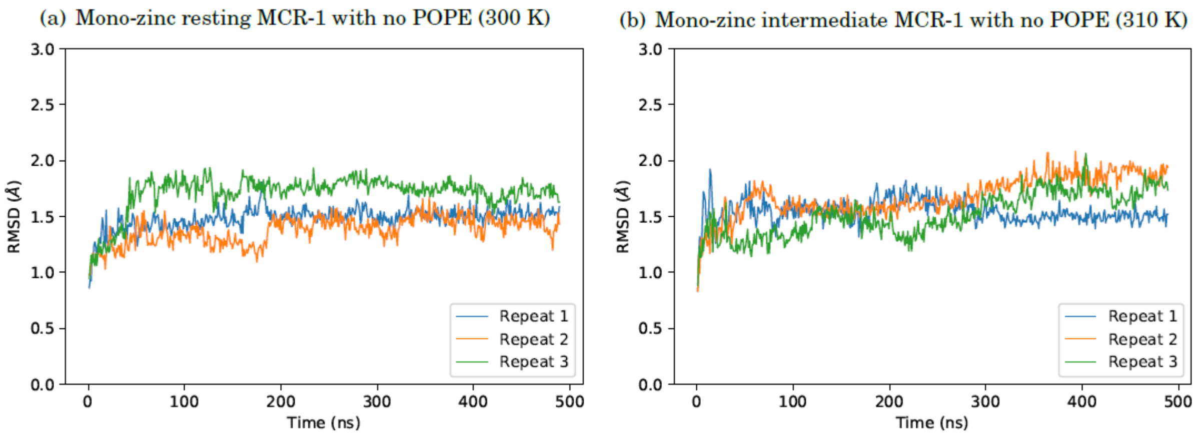

Figure S13. Time-Dependence of Cα RMSD Values for Unrestrained MD Simulations of MCR-1 at 300 and 310 K: MCR-1 Catalytic Domain. Panels show three repeat 500 ns simulations of two otherwise identical systems, run at 300 K (approximating room temperature) and 310 K (approximating body temperature). Both systems are mono-zinc full length MCR-1 with Thr285 unmodified and without bound POPE. RMSD calculations were performed after aligning trajectories to the catalytic domain (residues 219 - 541) of the initial homology model (Figure S1) built upon EptA (5FGN[3]).

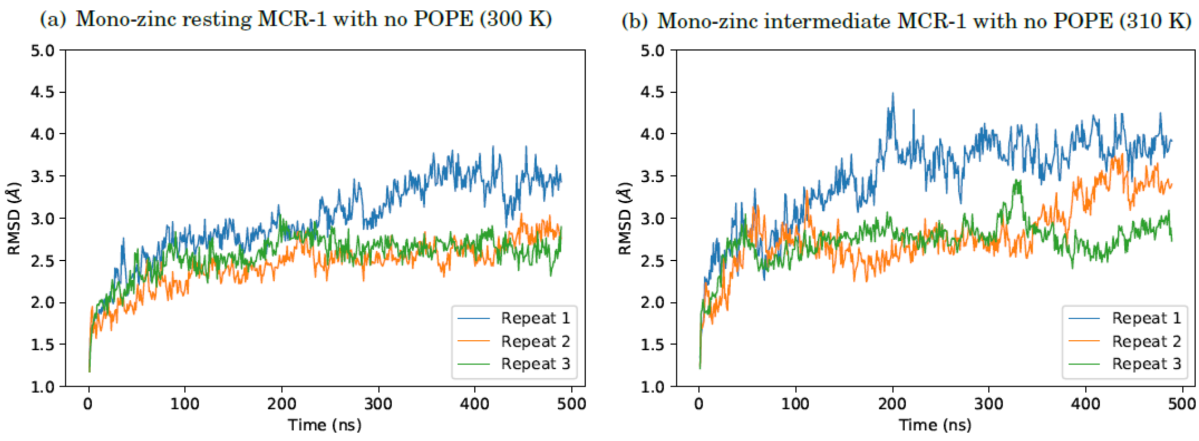

Figure S14. Time-Dependence of Cα RMSD Values for Unrestrained MD Simulations of MCR-1 at 300 and 310 K: MCR-1 Transmembrane Domain. Panels show three repeat 500 ns simulations of two otherwise identical systems, run at 300 K (approximating room temperature) and 310 K (approximating body temperature). Both systems are mono-zinc full length MCR-1 with Thr285 unmodified and without bound POPE. RMSD calculations were performed after aligning trajectories to the transmembrane domain (residues 11 - 218) of the initial homology model (Figure S1) built upon EptA (5FGN[3]).

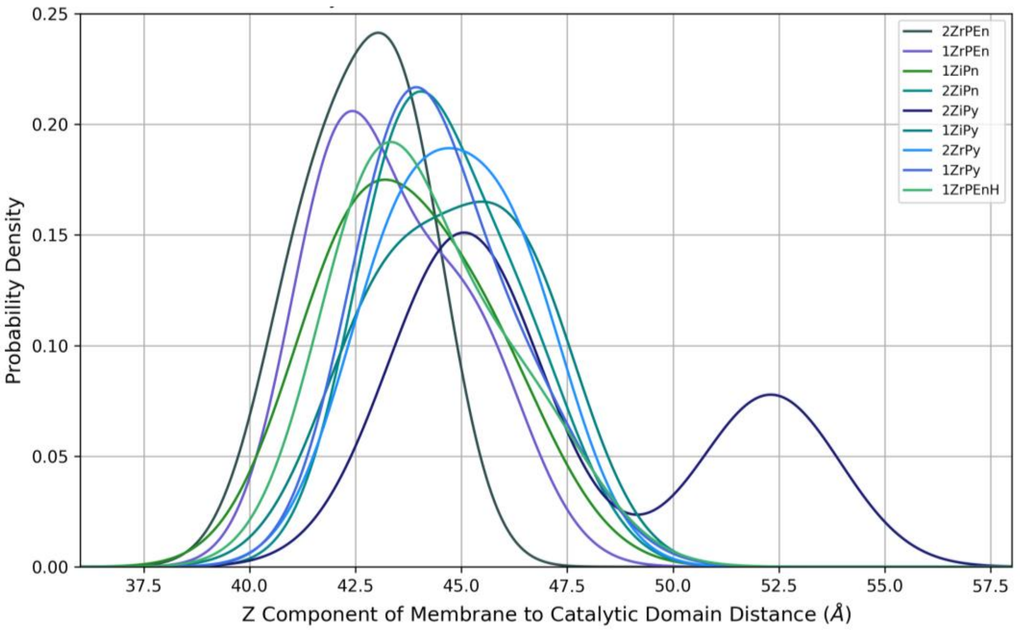

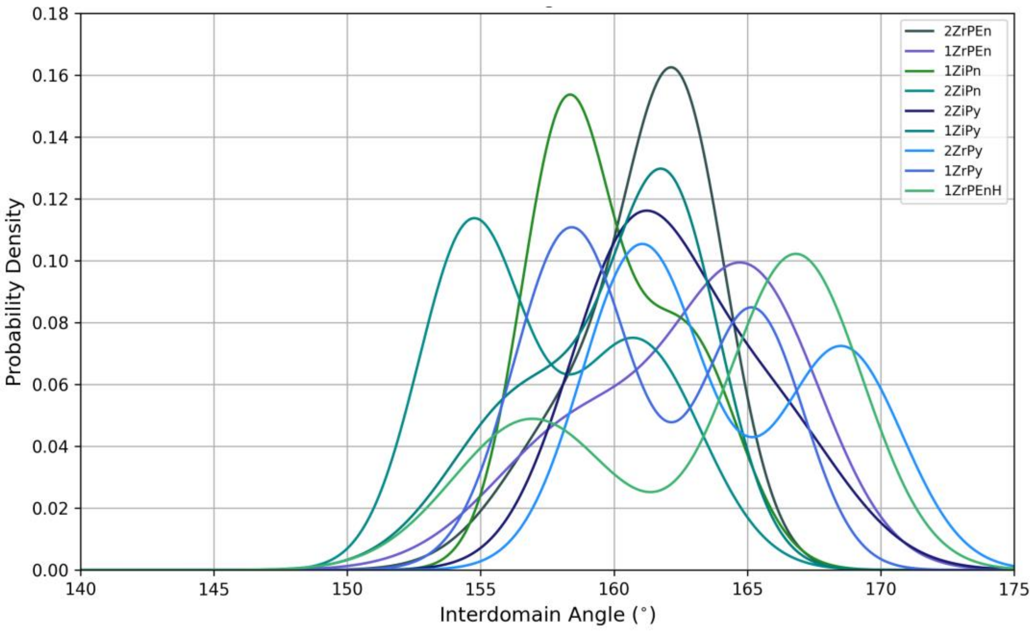

Figure S15. Distributions of Interdomain Distances/Angles for Unrestrained Simulations of Full-length MCR-1. Gaussian mixture models fitting bimodal distributions of (top) the Z-component of the membrane to the catalytic domain distance and (bottom) the interdomain angle (defined as the angle between the center-of-mass (COM) of the catalytic domain, the COM of the Zn1 co-ordinating residues (Glu246, Asp465, His466) and the COM of the transmembrane domain) for unrestrained simulations of MCR-1 (all replicates, sampling every ns over 50 – 500 ns). Fitting used sklearn.mixture within the scikit-learn[7] platform. Angle. Systems simulated are labelled as follows: “2ZrPEn” = di-zinc, unmodified Thr285, POPE-unbound; “1ZrPEn” = mono-zinc, unmodified Thr285, POPE-unbound; “1ZiPn” = mono-zinc, PEtN-modified Thr285, POPE-unbound; “2ZiPn” = di-zinc, PEtN-modified Thr285, POPE-unbound; “2ZiPy” = di-zinc, PEtN-modified Thr285, POPE-bound; “1ZiPy” = mono-zinc, PEtN-modified Thr285, POPE-bound; “2ZrPy” = di-zinc, unmodified Thr285, POPE-bound; “1ZrPy” = mono-zinc, unmodified Thr285, POPE-bound; “1ZrPEnH” = mono-zinc, unmodified Thr285, POPE-unbound, 310 K.

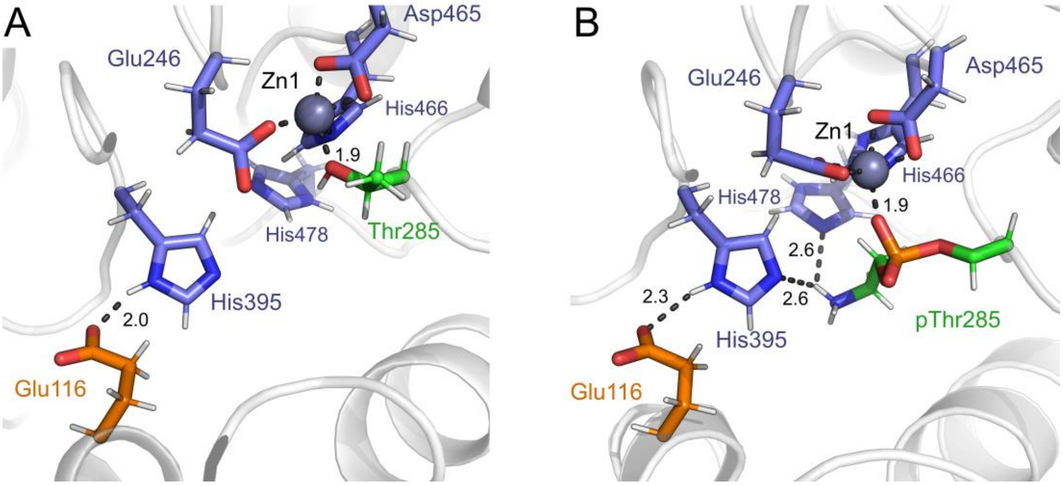

Figure S16. Active Site Interactions in Mono-zinc MCR-1. (A) Final frame from example MD simulation of mono-zinc MCR-1 with Thr285 unmodified and without substrate (POPE) in the active site. Note the hydrogen bond (2.0 Å) between His395 and Glu116. (B) Final frame from example MD simulation of mono-zinc MCR-1 with phosphoethanolamine (PEtN) covalently bound to Thr285 (pThr285) but without substrate (POPE) in the active site. Note interactions between PEtN phosphate and zinc (Zn1) ion (1.9 Å), and two hydrogen bonds between the PEtN amine tail and the "gating" histidine residues His395 and His478 (both 2.6 Å). Hydrogen and co-ordination bonds are shown as dashed lines.

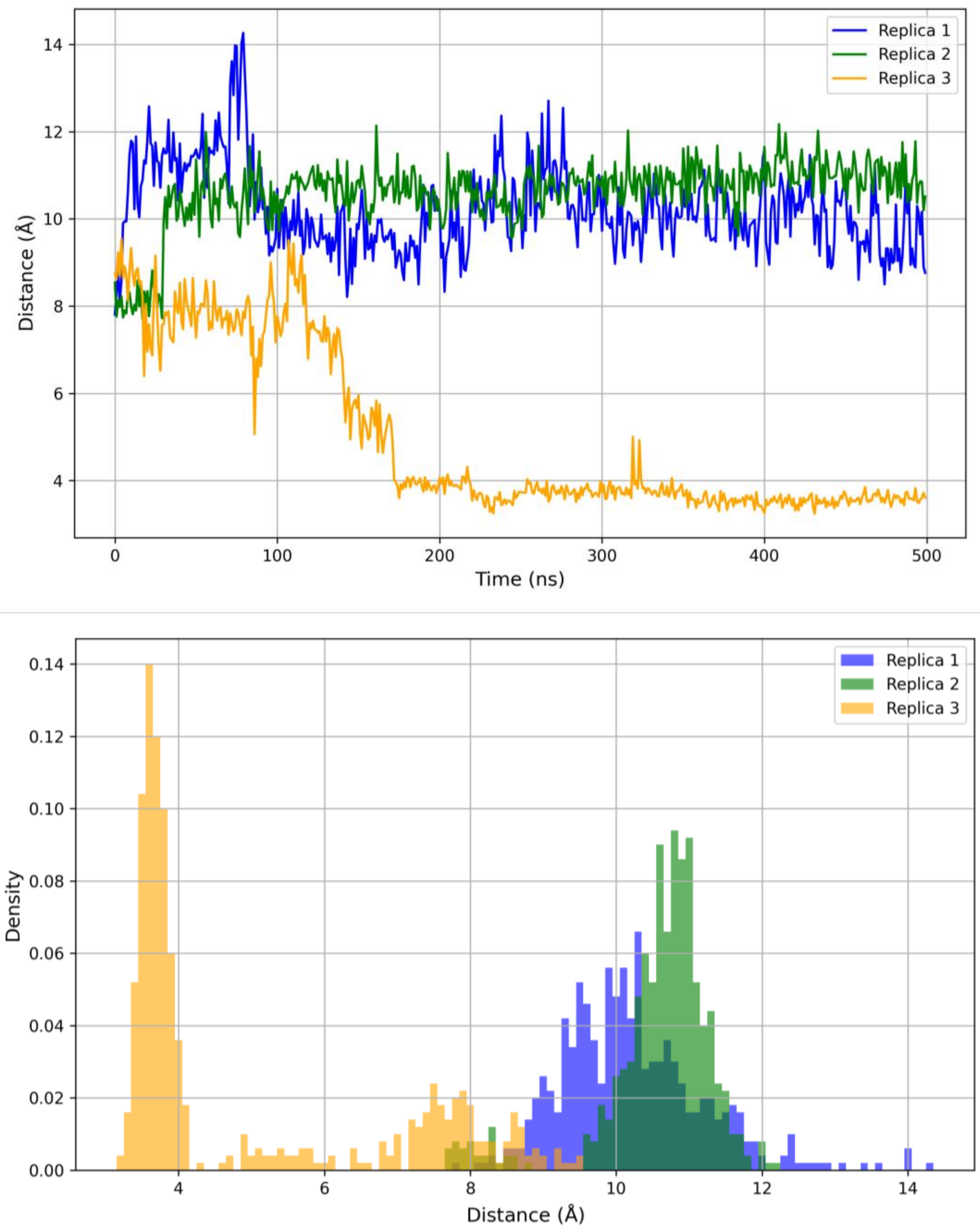

Figure S17. Interaction Between Thr285 and Bound POPE in Simulations of Mono-zinc MCR-1. (Top). distance between the phosphorus atom of the POPE headgroup and the Thr285 hydroxyl oxygen, monitored over time for all three repeat simulations (replicates 1 - 3) of mono-zinc POPE-bound unmodified MCR-1. (Bottom). Histogram of POPE P – Thr285 distances for all three replicate simulations of mono-zinc POPE-bound unmodified MCR-1 over the 50 - 500ns timeframe, values are rounded to the closest 0.1 Å.

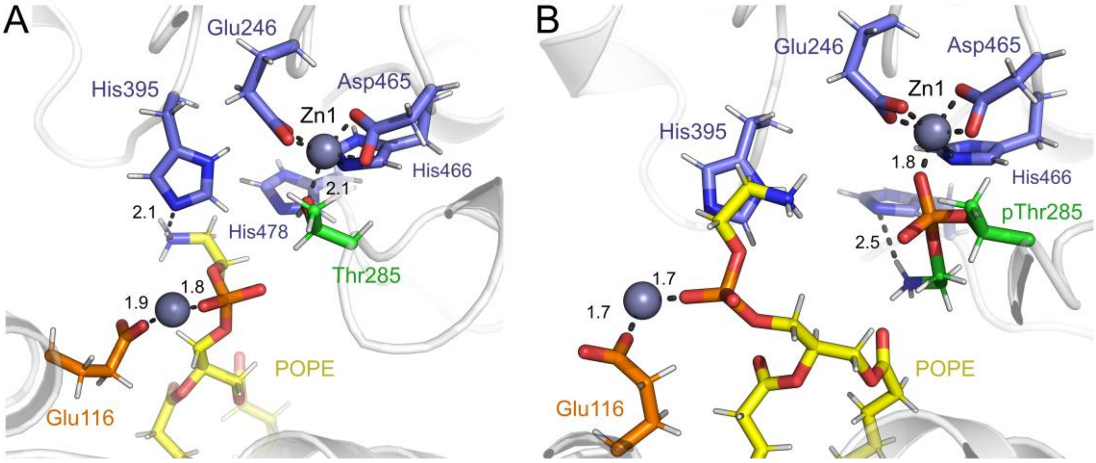

Figure S18. Unrestrained Simulations of di-Zinc MCR-1 with Bound POPE. (A) Final frame from example MD simulation of di-zinc MCR-1 with Thr285 unmodified and substrate (POPE) docked into the active site. Note stable coordinate bond (2.1 Å) between Thr285 hydroxyl and Zn1, dissociation of Zn2 from the His395/His478 binding site and interactions with POPE (1.8 Å) and Glu116 (1.9 Å), and hydrogen bond between PEtN amine to the gating histidine residue His478 (2.5 Å). (B) Final frame from example MD simulation of di-zinc MCR-1 with phosphoethanolamine (PEtN) covalently bound to Thr285 (pThr285) and substrate (POPE) docked into the active site. Note stable coordinate bond (1.8 Å) between PEtN phosphoryl oxygen and Zn1, while the. Zn2 readily dissociates from the Zn2 binding site and instead interacts with POPE (1.7 Å) and Glu116 (1.7 Å). Hydrogen and co-ordination bonds are shown as dashed lines.

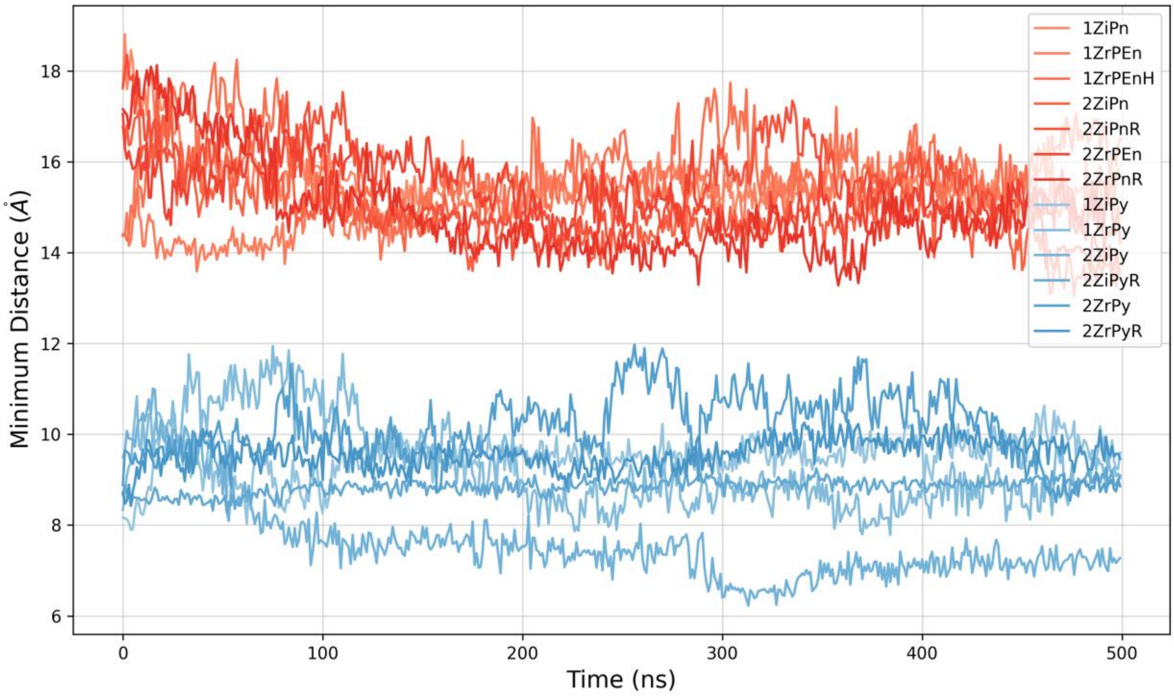

Figure S19. Time-dependence of Distance from Bound POPE to Active Site for MD Simulations of MCR-1. Time-dependence of the distance between the MCR-1 active site, defined as the center of mass of the residues bound to zinc in all simulations: Glu236, Asp455 and His456, and the closest atom of the PEtN head group of the closest POPE molecule. POPE bound simulations are shown in blue, and POPE-unbound in red, colors. Systems simulated are labelled as follows: “2ZrPEn” = di-zinc, unmodified Thr285, POPE-unbound; “1ZrPEn” = mono-zinc, unmodified Thr285, POPE-unbound; “1ZiPn” = mono-zinc, PEtN-modified Thr285, POPE-unbound; “2ZiPn” = di-zinc, PEtN-modified Thr285, POPE-unbound; “2ZiPy” = di-zinc, PEtN-modified Thr285, POPE-bound; “1ZiPy” = mono-zinc, PEtN-modified Thr285, POPE-bound; “2ZrPy” = di-zinc, unmodified Thr285, POPE-bound; “1ZrPy” = mono-zinc, unmodified Thr285, POPE-bound; “1ZrPEnH” = mono-zinc, unmodified Thr285, POPE-unbound, 310 K; “2ZrPnR” = restrained, di-zinc, unmodified Thr285, POPE-unbound; “2ZrPyR” = restrained, di-zinc, unmodified Thr285, POPE-bound; “2ZiPnR” = restrained, di-zinc, PEtN-modified Thr285, “2ZiPyR” = restrained, di-zinc, PEtN-modified Thr285, POPE-bound.

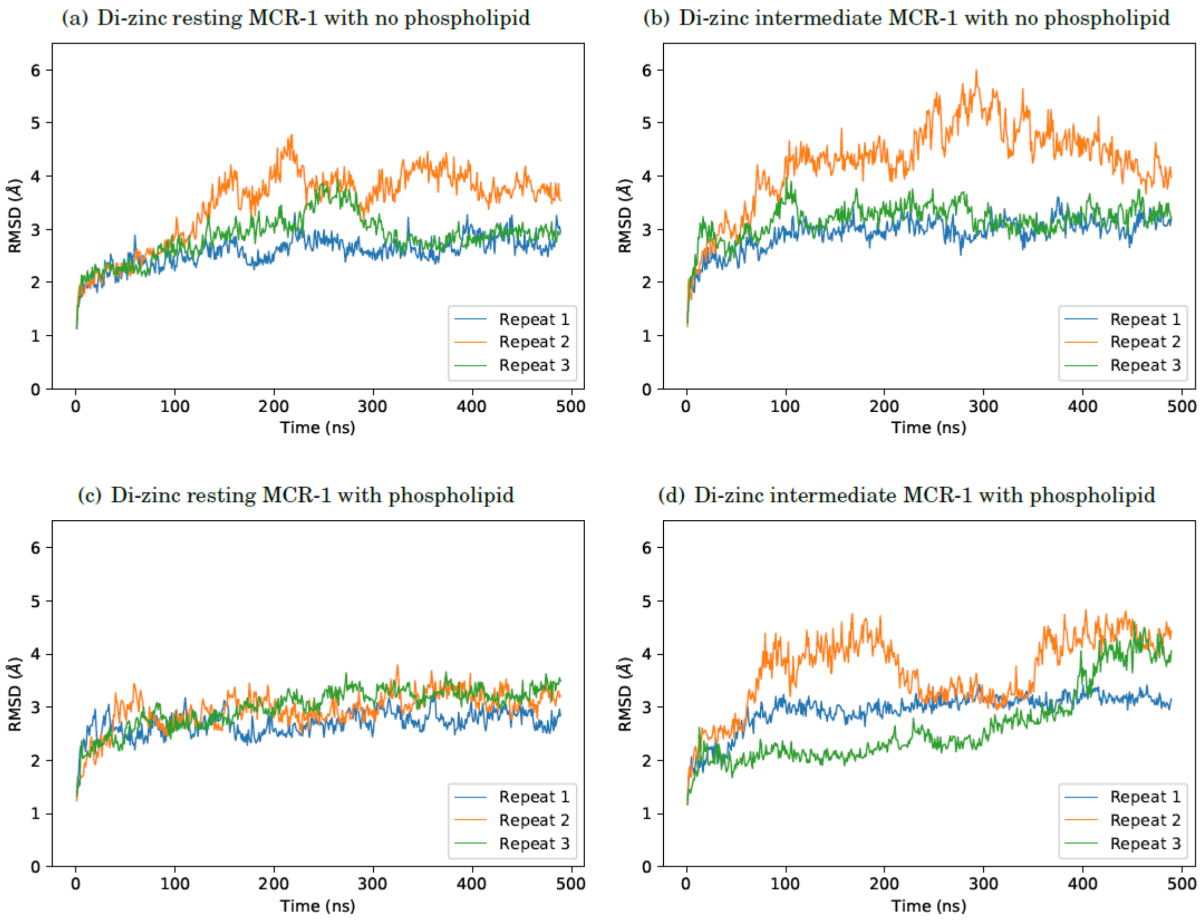

Figure S20. Time-Dependence of Cα RMSD Values for Restrained MD Simulations of di-Zinc MCR-1 at 300 K: Full-Length MCR-1. Panels show three 500 ns repeat simulations for four different systems, each with two zincs initially bound in the active site and distance and angle restraints applied throughout as described: (a) with Thr285 unmodified and without bound POPE; (b) with phosphoethanolamine covalently bound to Thr285 but without bound POPE; (c) with Thr285 unmodified and with bound POPE; (d) with phosphoethanolamine covalently bound to Thr285 and with bound POPE. All RMSD calculations were performed after aligning trajectories to the complete (residues 11 - 541) initial homology model built upon EptA (5FGN[3]).

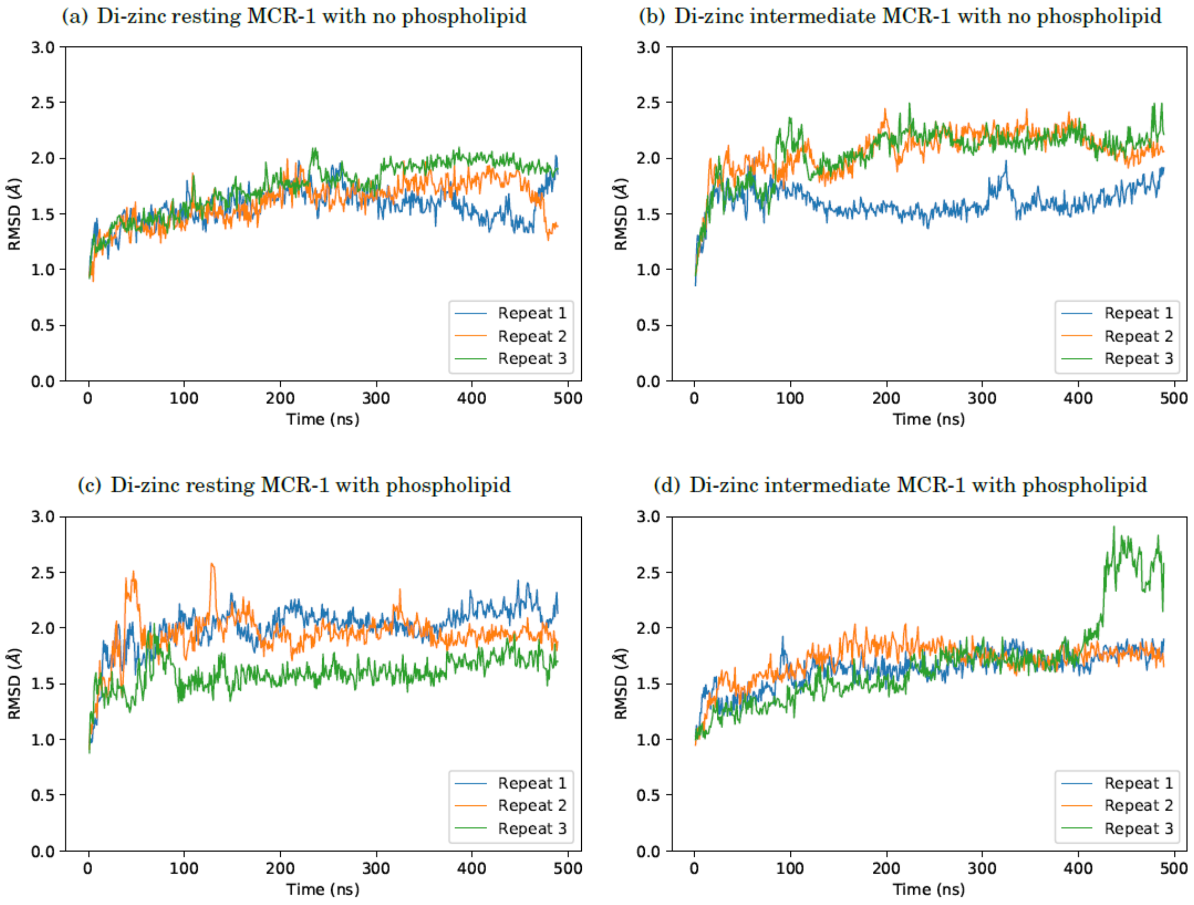

Figure S21. Time-Dependence of Cα RMSD Values for Restrained MD Simulations of di-Zinc MCR-1 at 300 K: MCR-1 Catalytic Domain. Panels show three 500 ns repeat simulations for four different systems, each with two zincs initially bound in the active site and distance and angle restraints applied throughout as described: (a) with Thr285 unmodified and without bound POPE; (b) with phosphoethanolamine covalently bound to Thr285 but without bound POPE; (c) with Thr285 unmodified and with bound POPE; (d) with phosphoethanolamine covalently bound to Thr285 and with bound POPE. RMSD calculations were performed after aligning trajectories to the catalytic domain (residues 219 - 541) of the initial homology model (Figure S1) based upon EptA (5FGN[3]).

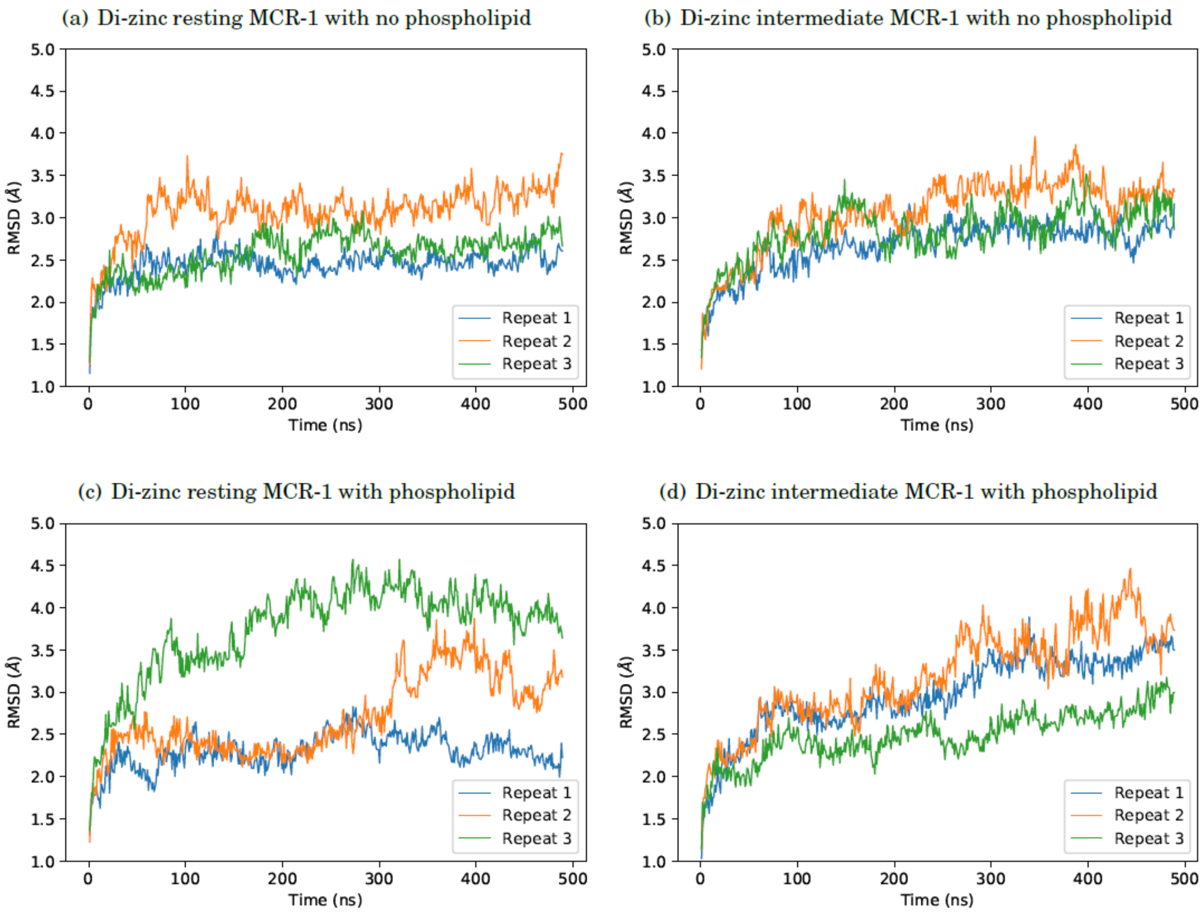

Figure S22. Time-Dependence of Cα RMSD Values for Restrained MD Simulations of di-Zinc MCR-1 at 300 K: MCR-1 Transmembrane Domain. Panels show three 500 ns repeat simulations for four different systems, each with two zincs initially bound in the active site and distance and angle restraints applied throughout as described: (a) with Thr285 unmodified and without bound POPE; (b) with phosphoethanolamine covalently bound to Thr285 but without bound POPE; (c) with Thr285 unmodified and with bound POPE; (d) with phosphoethanolamine covalently bound to Thr285 and with bound POPE. RMSD calculations were performed after aligning trajectories to the transmembrane domain (residues 11 - 218) of the initial homology model (Figure S1) built upon EptA (5FGN[3]).

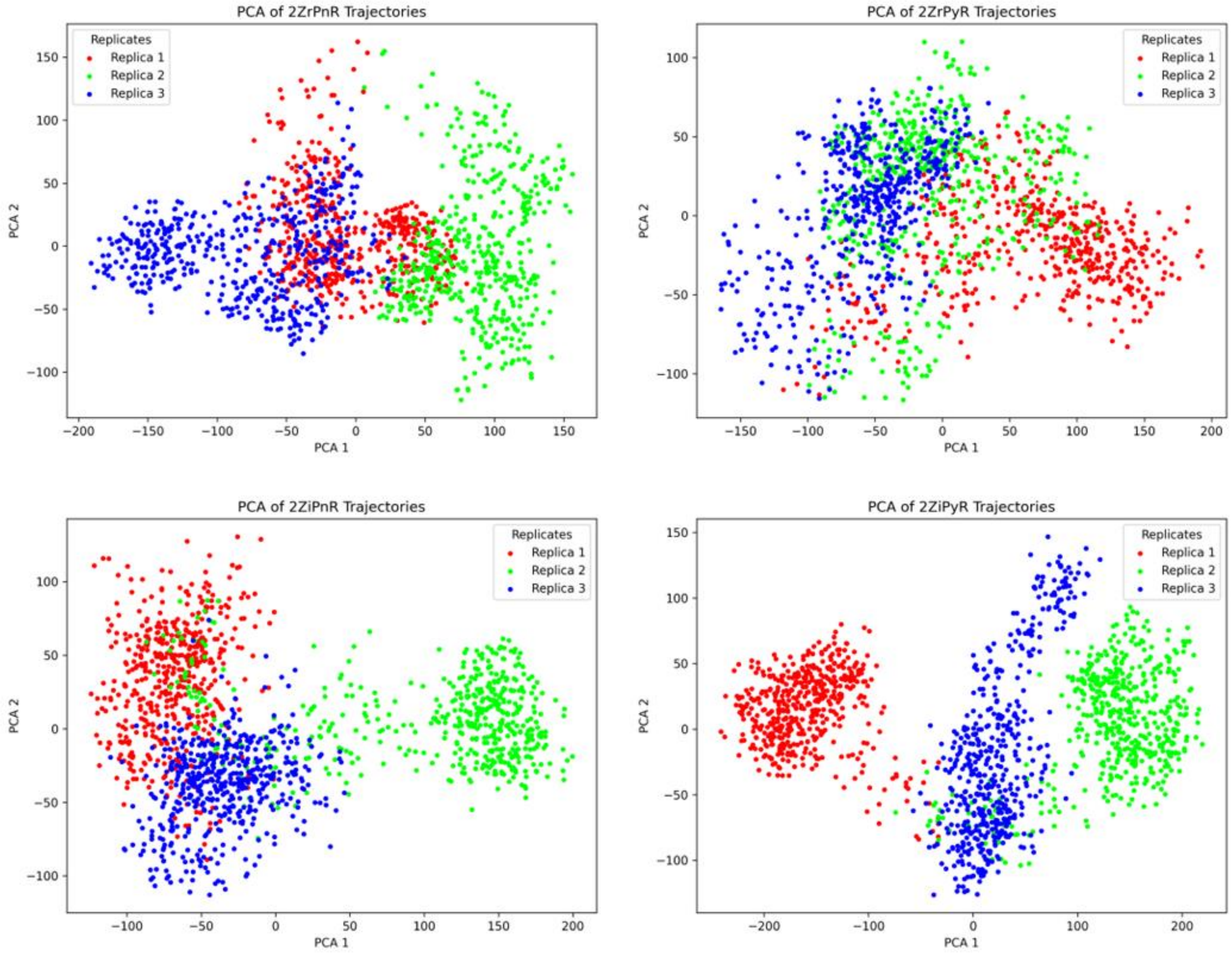

Figure S23. Principal Component Analysis (PCA) for Restrained Simulations of Full-length MCR-1 at 300 K. All three replicates for each system were combined before analysis such that they all shared a common subspace. Each trajectory was sampled once per nanosecond for the complete 500 ns of simulation (i.e. a total of 1500 frames). Analysis was carried out based on $C\alpha$ atom positions using the MDanalysis[5] PCA tool. Systems simulated are labelled as follows: "2ZrPnR" = restrained, di-zinc, unmodified Thr285, POPE-unbound; "2ZrPyR" = restrained, di-zinc, unmodified Thr285, POPE-bound; "2ZiPnR" = restrained, di-zinc, PEtN-modified Thr285, "2ZiPyR" = restrained, di-zinc, PEtN-modified Thr285, POPE-bound.

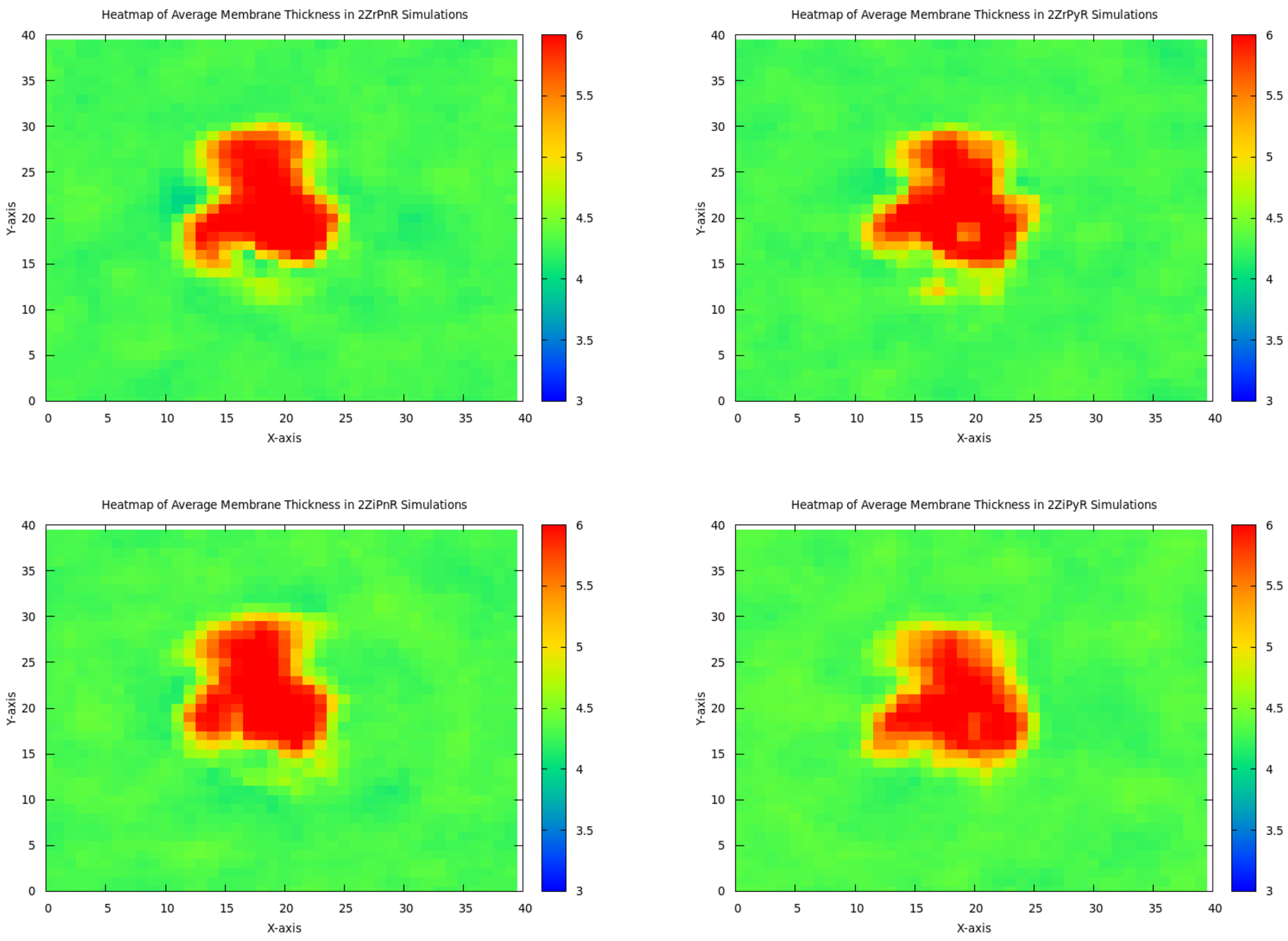

Figure S24. Measurements of Membrane Thickness for Restrained Simulations of MCR-1. GRIDMATMD[1] was used to measure membrane thickness. The membrane was divided into a 40 x 40 grid resulting in individual cells ~0.39 nm across. X and Y axes denote grid positions. Phosphate atoms of the lipid headgroup were used as the reference points for lipid positions at each time frame. Color bar denotes membrane thickness in nm. The height of the embedded MCR-1 protein (red) was set at 6.0 nm. Plots show average values from snapshots taken every 20 ns from 50 – 500 ns of triplicate simulations for each system. Systems simulated are labelled as follows: “2ZrPnR” = restrained, di-zinc, unmodified Thr285, POPE-unbound; “2ZrPyR” = restrained, di-zinc, unmodified Thr285, POPE-bound; “2ZiPnR” = restrained, di-zinc, PEtN-modified Thr285, “2ZiPyR” = restrained, di-zinc, PEtN-modified Thr285, POPE-bound.

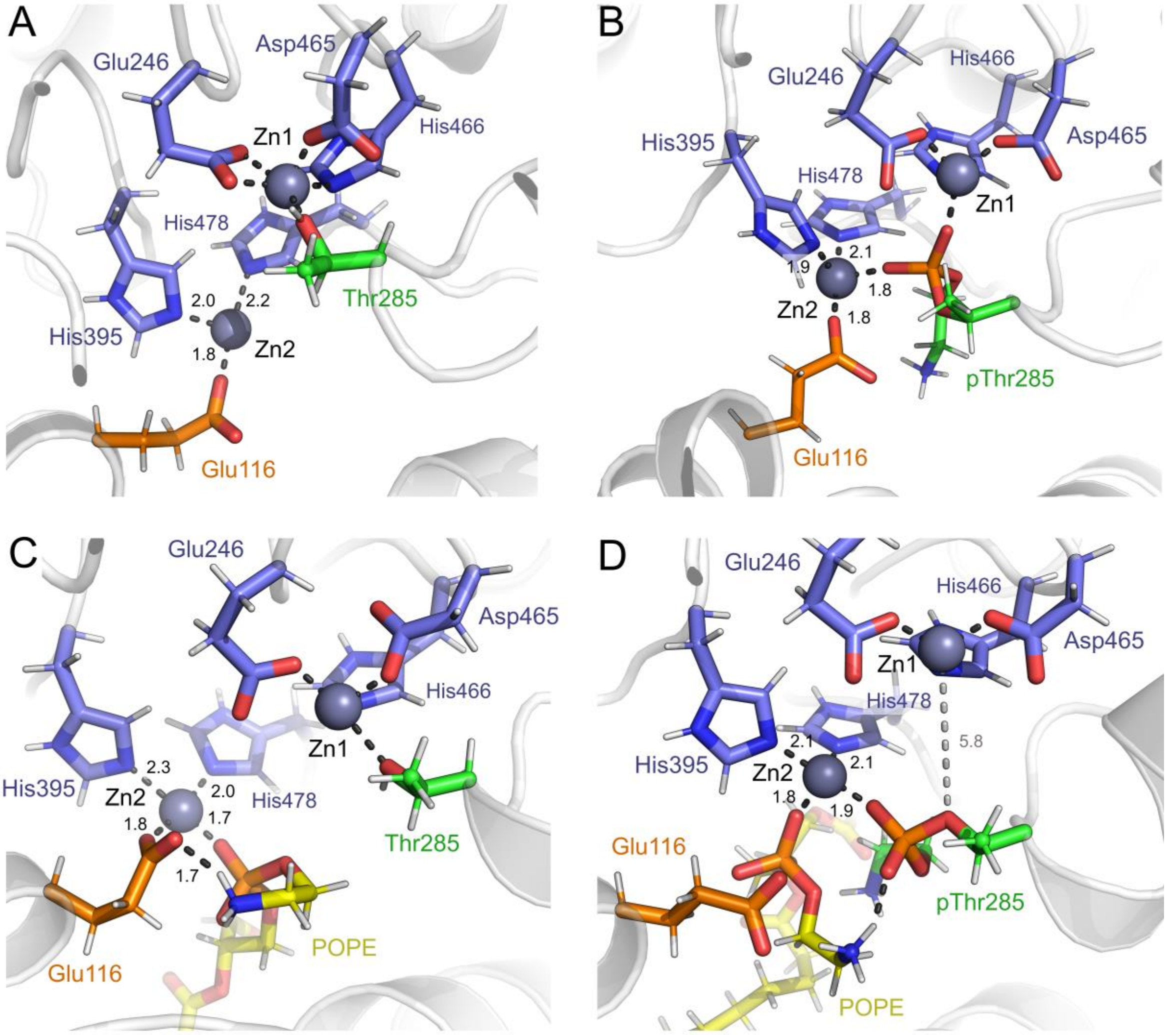

Figure S25. Active Sites of di-zinc MCR-1 from MD Simulations with Zinc Coordination Restrained. Snapshots of active sites from MD simulations of dizinc MCR-1 with restrained zinc coordination. Zn2 coordination distances are shown in Å. (A) Final frame from representative MD simulation of di-zinc MCR-1 with Thr285 unmodified and without bound POPE. All Zn coordination distances except for Glu116 OE1 - Zn2 (1.8 Å) were subject to harmonic restraints. (B) Final frame from representative MD simulation of di-zinc MCR-1 with phosphoethanolamine (PEtN) covalently bound to Thr285 and without bound POPE. All Zn coordination distances except for Glu116 OE1 - Zn2 (1.8 Å) and those involving the PEtN phosphoryl oxygen atoms (1.8 Å to Zn2, 1.9 Å to Zn1) were subject to harmonic restraints. (C) Final frame from representative MD simulation of di-zinc MCR-1 with Thr285 unmodified and with bound POPE. All zinc coordination interactions, except for those between Zn2 and Glu116 OE1 (1.8 Å) and POPE (1.7 Å) which formed spontaneously and were maintained throughout, were subject to harmonic restraints. (D) Final frame from representative MD simulation of di-zinc MCR-1 with phosphoethanolamine (PEtN) covalently bound to Thr285 and with bound POPE. All Zn coordination distances, except for the interactions between Zn2 and Thr285-bound PEtN (1.9 Å) and POPE (1.8 Å) which formed spontaneously and were maintained throughout, were subject to harmonic restraints. Hydrogen and co-ordination bonds are shown as dashed lines.

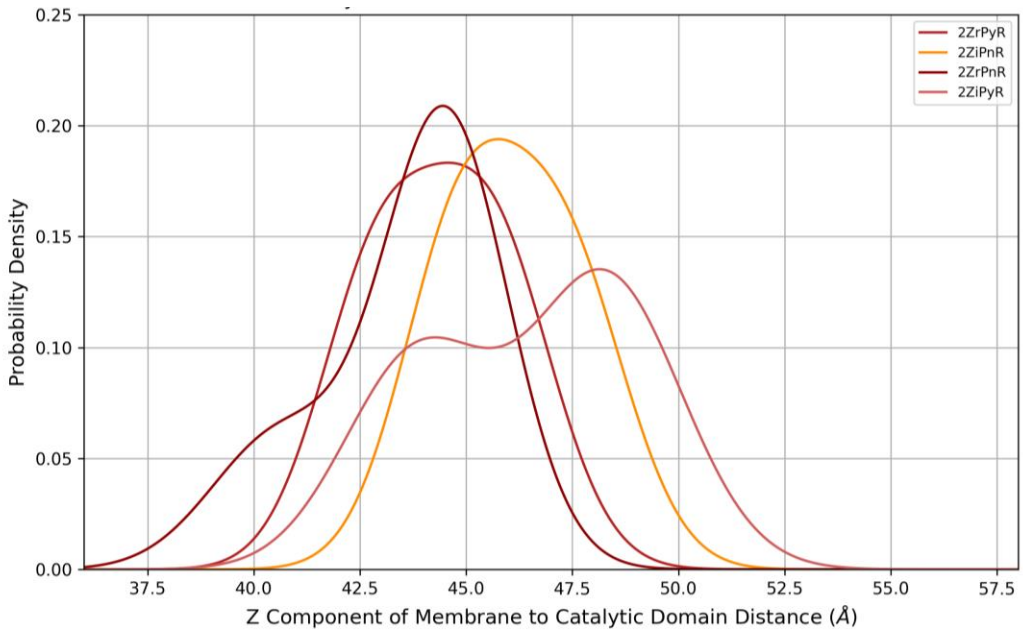

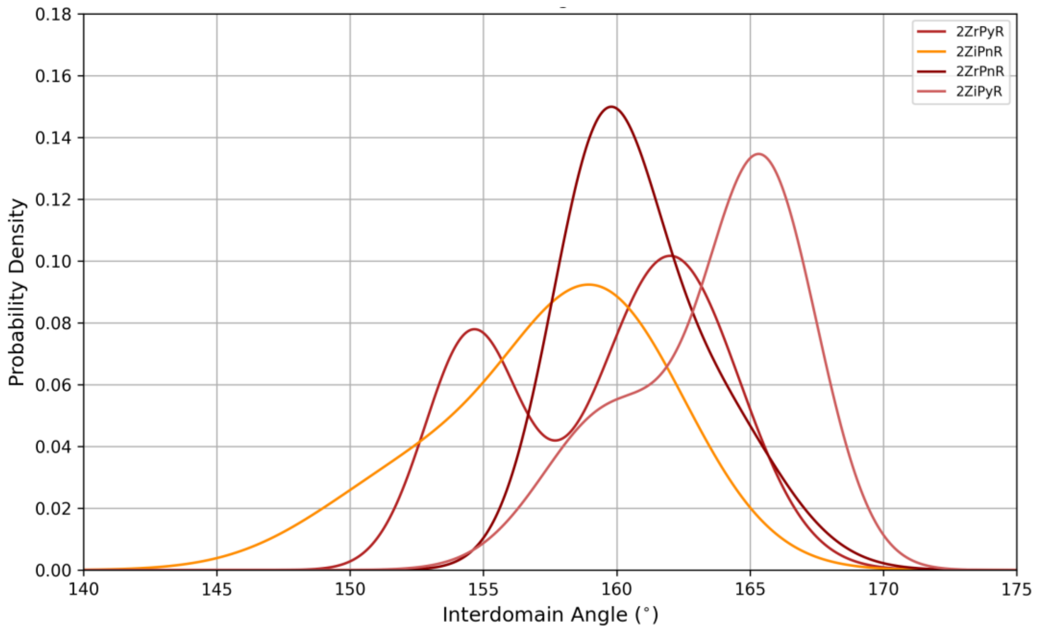

Figure S26. Distributions of Interdomain Distances/Angles for Restrained Simulations of Full-length MCR-1 at 300 K. Gaussian mixture models fitting bimodal distributions of (top) the Z-component of the membrane to the catalytic domain distance and (bottom) the interdomain angle (defined as the angle between the center-of-mass (COM) of the catalytic domain, the COM of the Zn1 co-ordinating residues (Glu246, Asp465, His466) and the COM of the transmembrane domain) for unrestrained simulations of MCR-1 (all replicates, sampling every ns over 50 – 500 ns). Fitting used sklearn.mixture within the scikit-learn[7] platform. Systems simulated are labelled as follows: "2ZrPnR" = restrained, di-zinc, unmodified Thr285, POPE-unbound; "2ZrPyR" = restrained, di-zinc, unmodified Thr285, POPE-bound; "2ZiPnR" = restrained, di-zinc, PEtN-modified Thr285, "2ZiPyR" = restrained, di-zinc, PEtN-modified Thr285, POPE-bound.

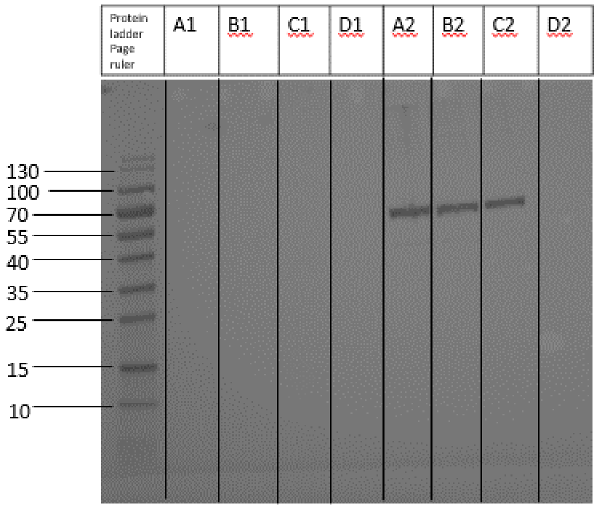

Figure S27. Expression Levels of Recombinant MCR-1 and Glu116 Mutants in *E. coli* DH5α. Figure shows Western blot (anti-*Myc* antibody) of whole-cell extracts of recombinant *E. coli* DH5α containing pBAD*/Myc*-HisC plasmids encoding A. wild-type MCR-1, B. MCR-1 Glu116Ala mutant, C. Glu116Leu mutant; or D. empty pBAD*/Myc*-HisC vector. Lanes labelled 1 denote uninduced cells, lanes labelled 2 are induced with 0.02 % arabinose. Black lines indicate positions of lanes on the gel.

Supporting References

(1) Allen, W. J.; Lemkul, J. A.; Bevan, D. R. GridMAT-MD: a grid-based membrane analysis tool for use with molecular dynamics. *J Comput Chem* **2009**, *30* (12), 1952-1958.
(2) Krissinel, E.; Henrick, K. Secondary-structure matching (SSM), a new tool for fast protein structure alignment in three dimensions. *Acta Crystallogr D Biol Crystallogr* **2004**, *60* (Pt 12 Pt 1), 2256-2268.
(3) Anandan, A.; Evans, G. L.; Condic-Jurkic, K.; O'Mara, M. L.; John, C. M.; Phillips, N. J.; Jarvis, G. A.; Wills, S. S.; Stubbs, K. A.; Moraes, I.; et al. Structure of a lipid A phosphoethanolamine transferase suggests how conformational changes govern substrate binding. *Proc Natl Acad Sci U S A* **2017**, *114* (9), 2218-2223.
(4) Abramson, J.; Adler, J.; Dunger, J.; Evans, R.; Green, T.; Pritzel, A.; Ronneberger, O.; Willmore, L.; Ballard, A. J.; Bambrick, J.; et al. Accurate structure prediction of biomolecular interactions with AlphaFold 3. *Nature* **2024**, *630* (8016), 493-500.
(5) Michaud-Agrawal, N.; Denning, E. J.; Woolf, T. B.; Beckstein, O. MDAnalysis: a toolkit for the analysis of molecular dynamics simulations. *J Comput Chem* **2011**, *32* (10), 2319-2327.
(6) Lythell, E.; Suardiaz, R.; Hinchliffe, P.; Hanpaibool, C.; Visitsatthawong, S.; Oliveira, A. S. F.; Lang, E. J. M.; Surawatanawong, P.; Lee, V. S.; Rungrotmongkol, T.; et al. Resistance to the "last resort" antibiotic colistin: a single-zinc mechanism for phosphointermediate formation in MCR enzymes. *Chem Commun (Camb)* **2020**, *56* (50), 6874-6877.
(7) Pedregosa, F.; Varoquaux, G.; Gramfort, A.; Michel, V.; Thirion, B.; Grisel, O.; Blondel, M.; Prettenhofer, P.; Weiss, R.; Dubourg, V.; et al. Scikit-learn: Machine Learning in Python. *J Mach Learn Res* **2011**, *12*, 2825-2830.
